# Antiseizure medications consumption in 73 countries and regions from 2012 to 2022: a longitudinal trend study

**DOI:** 10.1016/j.eclinm.2025.103558

**Published:** 2025-10-09

**Authors:** Adrienne Y.L. Chan, Andrew S.C. Yuen, Yingfen Hsia, Wallis C.Y. Lau, J. Helen Cross, Matthew C. Walker, Frank M.C. Besag, Anchor T.F. Hung, Noha Iessa, Neerja Chowdhary, Kenneth K.C. Man, Ian C.K. Wong

**Affiliations:** a Aston School of Pharmacy, Aston University, Birmingham, UK; b Centre for Safe Medication Practice and Research, Department of Pharmacology and Pharmacy, Li Ka Shing Faculty of Medicine, The University of Hong Kong, Hong Kong SAR, China; c Research Department of Practice and Policy, School of Pharmacy, University College London, London, UK; d Centre for Medicines Optimisation Research and Education, University College London Hospitals NHS Foundation Trust, London, UK; e UK Health Security Agency, London, UK; f Great Ormond Street Institute of Child Health, University College London, London, UK; g Great Ormond Street Hospital for Children NHS Foundation Trust, London, UK; h Department of Clinical and Experimental Epilepsy, UCL Queen Square Institute of Neurology, University College London, London, UK; i National Hospital for Neurology and Neurosurgery, University College London Hospitals NHS Foundation Trust, London, UK; j NIHR University College London Hospitals Biomedical Research Centre, London, UK; k East London Foundation NHS Trust, Bedfordshire, UK; l International Bureau for Epilepsy (Western Pacific), Hong Kong SAR, China; m Pharmacovigilance, Regulation and Safety, World Health Organisation, Geneva, Switzerland; n Department of Mental Health, Brain Health and Substance Use, World Health Organisation, Geneva, Switzerland; o School of Pharmacy, Medical Sciences Division, Macau University of Science and Technology, Taipa, Macau SAR, China; p Laboratory of Data Discovery for Health (D^2^4H), Hong Kong Science Park, Hong Kong SAR, China

**Keywords:** Antiseizure medications, Valproate, Valproic acid, Global health, Drug consumption

## Abstract

**Background:**

International trends in antiseizure medication use across countries from different geographical regions and income levels remain underexplored. Valproic acid (valproate) use has raised concerns due to its teratogenic risks, with World Health Organisation (WHO) guidelines recommending lamotrigine or levetiracetam as first-line therapy for epilepsy in women and girls of childbearing potential, advising against valproate use in this population. This study aimed to assess multinational trends in antiseizure medication (ASM) consumption from 2012 to 2022 in the context of evolving policy and regulatory actions.

**Methods:**

In this longitudinal trend study, we used pharmaceutical sales data of antiseizure medications from the IQVIA-Multinational Integrated Data Analysis System (MIDAS) between January 1, 2012 and December 31, 2022, covering 73 countries/regions. The list of ASMs included in this study was based on the Anatomical Therapeutic Chemical (ATC) Classification, N03A. We obtained the mid-year national/regional population estimates of each country from the United Nations Population Division and the total epilepsy (active idiopathic and secondary epilepsy) population from the Global Burden of Disease Collaborative Network. 41 high-income, 20 upper-middle income and 12 lower-middle-income countries/regions were included in this study. Antiseizure medication consumption rate was expressed as defined daily doses per 10,000 inhabitants per day (DDD/TID). Linear mixed models were used to estimate multinational, regional, and income-level trends in consumption over time.

**Findings:**

Multinational antiseizure medication consumption increased throughout the study period, with an average annual percentage change of +2·58% (95% CI +1·85% to +3·32%), rising from 40·96 DDD/TID (31·94–52·52) in 2012 to 52·87 DDD/TID (42·17–66·27) in 2022. The highest change in consumption was in South-eastern Asia (+5·20%, +3·41% to +7·03%), followed by Western Asia (+4·70%, +0·58% to +8·99%) and Southern Asia (+3·80%, +1·52% to +6·14%). Newer generation antiseizure medications such as levetiracetam (+21·72%, +13·86% to +30·11%) and lamotrigine (+7·48%, +6·34% to +8·63%) showed growth in consumption, while consumption of older medications such as phenobarbital (−2·85%, −9·50% to +4·29%), phenytoin (−11·19%, −17·58% to −4·30%), and carbamazepine (−1·09%, −1·95% to −0·23%) declined. In 2022, the consumption rate of high-income countries (88·36 DDD/TID, 71·69–108·90) was more than four times of lower-middle-income countries (15·63 DDD/TID, 8·75–27·91). Valproate remained the most widely used antiseizure medication globally (10·93 DDD/TID, 8·68–13·77) in 2022, with stronger growth observed in lower- (+4·24%, +1·73% to +6·81%) and upper-middle-income (+3·10%, +0·95% to +5·29%) countries, compared with high-income countries (+0·86%, −0·07% to +1·79%).

**Interpretation:**

Multinational antiseizure medication use increased between 2012 and 2022, particularly for newer medications like levetiracetam and lamotrigine. Disparities in access to antiseizure medications across countries of varying income levels persist, with valproate consumption remaining predominant. This underscores the urgent need to align prescribing practice with safety guidelines, in order to optimise patient outcomes. Since patient-level characteristics are not available in IQVIA-MIDAS, further research is warranted to examine consumption rates across different population groups.

**Funding:**

This study is partially supported by the Laboratory of Data Discovery for Health (D^2^4H) funded by AIR@InnoHK, administered by Innovation and Technology Commission of the Government of Hong Kong Special Administrative Region.

**Research in context**

**Evidence before this study**

We searched PubMed on June 27, 2025 for studies published between January 1, 2010, and June 27, 2025, with the following terms: ((antiseizure) OR (antiepileptic) OR (anticonvulsant)) AND ((medication) OR (drug)) AND ((consumption) OR (utilisation) OR (use) OR (sales) OR (MIDAS)) AND ((trend) OR (longitudinal) OR (temporal) OR (“time series”)) AND ((global) OR (international) OR (multinational) OR (cross-national) OR (country) OR (countries) OR (region∗) OR (worldwide)). This search returned 500 articles and 21 of them quantified utilisation or consumption of antiseizure medications (ASMs). However, all these previous studies were restricted to single countries, to sub-populations (pregnant women or children), or a few countries from European Union, North America and Australia. Most of them focused on high-income settings and none examined ASM consumption at a global or multinational scale. Consequently, the worldwide patterns and inequalities in ASM consumption, particularly in low- and middle-income countries, have remained understudied.

**Added value of this study**

This study analysed ASM consumption trend from 73 countries/regions that together account for approximately 77% of the world's population, using standardised data from a single source. It is the first to quantify worldwide access disparities in ASMs, showing the gap between high-income countries/regions and lower-middle-income settings. It visualises the rise of newer agents such as levetiracetam and lamotrigine which are steadily replacing older ASMs. It also tracks valproate consumption across diverse health systems, revealing diverging trends despite recent warnings and pregnancy prevention programmes.

**Implications of all the available evidence**

Consumption of ASMs has risen steadily over the past decade, signalling broader availability, yet national consumption rates remain highly uneven. Access gaps are particularly pronounced in many lower-middle-income countries, where utilisation still lags far behind their high-income counterparts. Regulatory warnings and pregnancy-prevention programmes have not produced a sustained worldwide decline in valproate use. National authorities, particularly in lower-income settings, should enhance efforts to improve equitable access to ASMs that are listed in the World Health Organisation model list of essential medicines, to ensure effective epilepsy control. Our study findings also offer a baseline for evaluating the impact of future interventions related to the use of valproate.

## Introduction

Antiseizure medications (ASMs), previously known as anticonvulsants or antiepileptics, are a class of medications primarily used for the treatment of epilepsy, with the aim of reducing the frequency and severity of seizures. Their mechanisms of action are diverse, including the modulation of sodium, calcium, or potassium ion channels, enhancement of gamma-aminobutyric acid-mediated inhibitory action, and attenuation of excitatory neurotransmitters.^1^ First-generation ASMs, such as phenytoin, carbamazepine, and valproic acid (or valproate), have been used for decades. More recently, second-generation ASMs including lamotrigine, levetiracetam, and topiramate, and third-generation such as lacosamide, brivaracetam or perampanel have been introduced with the intention to improve pharmacokinetics, fewer drug interactions, and broader therapeutic profiles.^1^ Beyond epilepsy, some ASMs are also indicated for other neurologic or psychiatric conditions, such as bipolar disorder, migraine and neuralgia.^1^

ASMs carry distinct pharmacological properties and sometimes serious adverse effect profiles. For instance, phenobarbital is notable for its abuse potential and pronounced sedation.^2^ Phenytoin demands careful dose titration and routine serum monitoring because of its narrow therapeutic window and complex, non-linear pharmacokinetics.^2^ Carbamazepine can precipitate life-threatening cutaneous reactions such as Stevens–Johnson syndrome,^2^ whereas topiramate is linked to cognitive slowing and executive function decline.^2^ In addition, including valproate, all the above ASMs are associated with increased risk of teratogenicity when used during pregnancy to a varying extent.^2^

Among all ASMs, valproate is one of the most effective broad-spectrum agents, widely prescribed for epilepsy, bipolar disorder, and migraine.^3^ However, its use in women and girls of childbearing potential has drawn increasing scrutiny due to its strong association with teratogenicity and neurodevelopmental harm.^4^ Accumulating evidence has linked in utero valproate exposure with congenital malformations, reduced intelligence quotient (IQ), and a higher risk of autism spectrum disorders.^5^^,^^6^ These risks prompted regulatory actions worldwide. The European Medicines Agency (EMA) strengthened warnings for use of valproate in girls and women in November 2014,^7^^,^^8^ followed by the implementation of the Pregnancy Prevention Programme in 2018,^9^^,^^10^ which restricts valproate use in women unless no suitable alternative exists. In the UK, the Medicines and Healthcare products Regulatory Agency (MHRA) has progressively tightened prescribing guidance since 2015,^11^ and most recently even issued a warning regarding valproate use in men around the time of conception,^11^^,^^12^ aligning with EMA recommendations that valproate should be initiated and supervised by a specialist in male patients.^13^ In the US, the Food and Drug Administration (FDA) contraindicated valproate for migraine prevention during pregnancy in 2013^14^ and added boxed warnings for its teratogenicity and risk of cognitive impairment. The World Health Organisation (WHO) also updated its Mental Health Gap Action Programme (mhGAP) guidelines in 2023, explicitly advising against the use of valproate in women and girls of childbearing age due to the associated high risks.^15^ Despite these measures, recent studies continue to report persistent or even increasing use of valproate in some populations, raising concerns related to the current prescribing practice across different countries.^16^^,^^17^

Previous studies on ASM utilisation have largely been limited to specific countries, regions, or drug classes, and have largely been conducted in high-income settings,^18^, ^19^, ^20^, ^21^ which may limit their generalisability to lower-middle-income countries. To address this gap, our study aimed to examine the global trends in ASM consumption across 73 countries and regions from 2012 to 2022. By focussing on both overall ASM use and trends specific to valproate, this research may inform international policy discussions about ASM use and specifically guide further risk minimisation measures to promote safe and evidence-based prescribing practices.

## Methods

### Study design and data source

In this longitudinal trend study, we utilised multinational sales data from the IQVIA-Multinational Integrated Data Analysis System (MIDAS) database. IQVIA is a company specialising in healthcare analytic data. MIDAS captures multinational data on sales volumes of pharmaceutical products from various distribution channels including manufacturers, wholesalers, hospitals, and retail pharmacies, and applies international standardisation to allow comparisons of national sales volumes. The average national coverage of MIDAS data was 88%.^22^ For countries where the MIDAS database did not have 100% sector coverage, adjustments were made by IQVIA to estimate the total sales volume based on knowledge of the market share of participating wholesalers and retail or hospital pharmacies.^23^ The MIDAS database has been validated against external data sources^24^ and used as a proxy to evaluate multinational consumption of different classes of medications.^25^, ^26^, ^27^, ^28^ With a similar approach in previous studies, we utilised the sales data to investigate the consumption of the medication by patients in each country. The MIDAS database does not contain patient-level data; thus, no information on patient demographics, including race or ethnicity, was available and institutional review board approval was not required. We obtained the mid-year national/regional population estimates of each country from the United Nations Population Division^29^ and the total epilepsy (active idiopathic and secondary epilepsy) population from the Global Burden of Disease Collaborative Network.^30^ The definition for epilepsy was based on the International League Against Epilepsy (ILAE) guidelines for epidemiologic studies on epilepsy,^31^ which defined an epilepsy case as someone with an active, recurrent condition of epileptic seizures (two or more) unprovoked by an immediate cause and who has had at least one epileptic seizure in the past 5 years regardless of antiseizure drug treatment.

### Data inclusion

Data on the sales of ASMs between January 1, 2012 and December 31, 2022 were extracted from 73 countries and regions in the IQVIA-MIDAS database. The list of ASMs included in this study was based on the Anatomical Therapeutic Chemical (ATC) Classification, N03A, and is available in Supplementary Table S1. Other treatment options which are not under N03A, such as diazepam and midazolam, were not included in the study. The characteristics of included countries and their respective database market coverage are presented in Supplementary Table S2. The included countries were divided into the following areas: Northern America, Central and Southern America and the Caribbean, Northern Europe, Eastern Europe, Southern Europe, Western Europe, Oceania, Eastern Asia, Central Asia, South-eastern Asia, Southern Asia, Western Asia, Northern Africa, and Southern Africa, based on their geographical regions according to United Nations' “*Standard Country or Area Codes for Statistical Use*”.^32^

### Statistical analysis

The main outcome metric was the rate of ASM consumption, expressed as the defined daily dose (DDD) per ten-thousand inhabitants per day (DDD/TID). DDD is the assumed average maintenance dose per day for a drug used for its main indication in adults and was only available for single-molecule products. As such, DDD for combination products was converted from a standard unit (defined as a single tablet, capsule, or ampoule/vial or 5 mL oral solution/suspension), formulation, with their respective drug ingredients mapped to the ATC/DDD Index developed by the WHO Collaborating Centre for Drug Statistics Methodology (Supplementary Table S1).^33^ Where the strength or formulation of the product was missing, they were imputed based on the respective information of the most-sold product of the same drug.^22^

At the national level, consumption rates in DDD/TID were calculated with a 95% confidence interval (CI) estimated by the Poisson method.^34^ The multinational and regional consumption levels were computed by pooling the estimates from individual countries using a random-effects model.^35^ The time trends of ASM consumption were evaluated at multinational, regional, and national levels across the study period. At the national level, the average annual percentage change in DDD/TID with 95% CI was estimated using a linear regression model, with log-transformed consumption in DDD/TID as the dependent variable and year as the independent variable. The multinational and regional trend changes were estimated using linear mixed models, controlling for within-country correlations and accounting for first-order autocorrelation between years.^26^ We further stratified the sales data based on country income levels (i.e., lower-middle income, upper-middle income, and high income according to the 2022 World Bank income classification)^36^ to investigate how consumption trends varied with country income levels. To investigate whether the prevalence of epilepsies affected the study findings, we performed a sensitivity analysis substituting the national population with the epilepsy population from the Global Burden of Disease (GBD) Study 2021^30^ and limited the study period to 2012–2021 where the most recent GBD data were available. The consumption rates were calculated as the DDD per hundred patients with epilepsy per day (DDD/HED), following the analytical methods in the main analyses. A 95% CI not overlapping with the null was considered statistically significant. All analyses were conducted using Statistical Analysis System (SAS) v9·4 (SAS Institute, Cary, NC, USA) and R Foundation for Statistical Computing version 3·6·0 (Vienna, Austria). This study followed the Strengthening the Reporting of Observational Studies in Epidemiology (STROBE) reporting guideline.^37^

### Role of the funding source

The funder of this study had no involvement in study design, data collection, data analysis, data interpretation, or writing of the report. AYLC, ASCY, KKCM, and ICKW had full access to the study data and held final responsibility for the decision to submit the manuscript for publication.

## Results

**Fig. 1:**
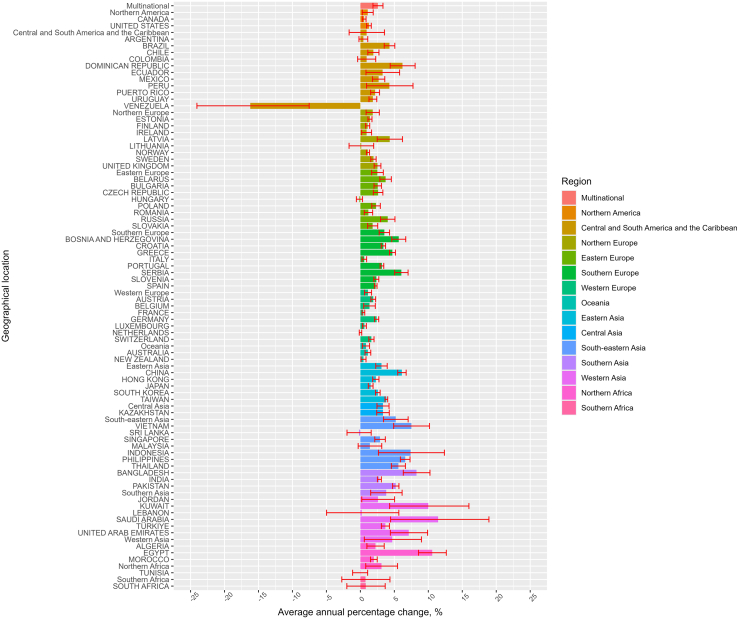
**Average annual percentage change of antiseizure medication consumption.** Different colours indicate the geographical regions of the included countries/regions, while the error bars represent the 95% CI for the average annual percentage change between 2012 and 2022.

**Fig. 2:**
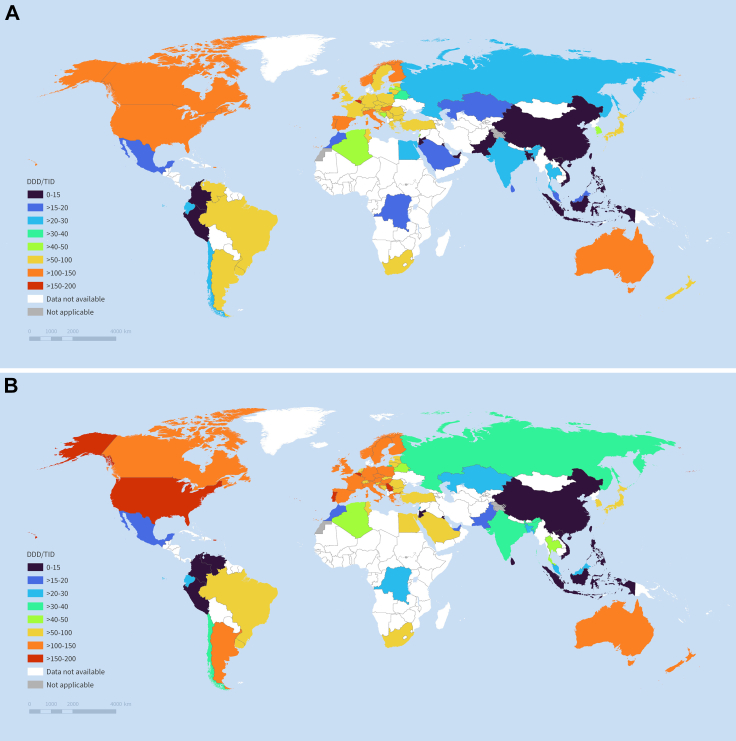
**A. Antiseizure medication consumption in DDD/TID in 2012. B. Antiseizure medication consumption in DDD/TID in 2022.** Different colours represent the different levels of DDD/TID across the included countries/regions. DDD/TID = Defined daily dose per 10,000 inhabitants per day. For DDD/TID category thresholds, see the bottom-left legend; both maps use the same breaks and palette. Please refer to Table 1 for the DDD/TID in each country/region. The designations employed and the presentation of the material in this publication do not imply the expression of any opinion whatsoever on the part of WHO concerning the legal status of any country, territory, city or area or of its authorities, or concerning the delimitation of its frontiers or boundaries. Dotted and dashed lines on maps represent approximate border lines for which there may not yet be full agreement.

Among the 73 countries/regions, representing approximately 77% of the global population, multinational ASM consumption rate recorded an increasing trend from 2012 to 2022 (Table 1, Fig. 1, Supplementary Figure S1) with an average annual percentage change of +2·58% (95% CI +1·85% to +3·32%), from 40·96 DDD/TID (95% CI 31·94–52·52) in 2012 to 52·87 DDD/TID (42·17–66·27) in 2022 (Table 1). Across included countries/regions, ASM consumption rate mostly increased from 2012 to 2022 (Table 1 and Fig. 1). Average annual percentage change in consumption was the highest in South-eastern Asia (+5·20%, +3·41% to +7·03%), followed by Western Asia (+4·70%, +0·58% to +8·99%) and Southern Asia (+3·80%, +1·52% to +6·14%). In 2022, pooled ASM consumption rates were the highest in Northern America (143·55 DDD/TID, 12·24–1683·59), followed by Southern Europe (124·17 DDD/TID, 99·76–154·56) and Western Europe (109·52 DDD/TID, 88·02–136·27). ASM consumption rate was lowest in South-eastern Asia (13·56 DDD/TID, 4·61–39·92) (Table 1 and Fig. 2).

**Table 1:** Multinational, regional, and national levels of antiseizure medication consumption rates in 2012 and 2022 and average annual percentage change.

Countries/regions	DDD/TID in 2012 (95% CI)^a^	DDD/TID in 2022 (95% CI)^a^	Average annual percentage change (%, 95% CI)^b^	P value
**Multinational**	40·96 (31·94–52·52)	52·87 (42·17–66·27)	+2·58 (+1·85 to +3·32)	<0·0001
**Northern America**	128·87 (19·15–867·10)	143·55 (12·24–1683·59)	+1·08 (+0·29 to +1·89)	0·010
Canada	110·92 (110·90–110·94)	118·27 (118·25–118·28)	+0·56 (+0·29 to +0·84)	0·0012
United States	149·73 (149·72–149·74)	174·25 (174·24–174·25)	+1·26 (+0·92 to +1·61)	<0·0001
**Central and South America and the Caribbean**	31·87 (16·65–60·99)	34·90 (17·80–68·42)	+0·91 (−1·66 to +3·55)	0·49
Argentina	95·86 (95·84–95·87)	101·09 (101·07–101·10)	+0·44 (−0·21 to +1·09)	0·16
Brazil	50·70 (50·70–50·71)	76·13 (76·13–76·14)	+4·29 (+3·51 to +5·07)	<0·0001
Chile	27·59 (27·58–27·60)	34·37 (34·36–34·39)	+1·91 (+1·10 to +2·73)	0·0005
Colombia	7·75 (7·74–7·75)	7·86 (7·86–7·87)	+0·92 (−0·40 to +2·26)	0·15
Dominican Republic	16·42 (16·40–16·43)	26·72 (26·70–26·74)	+6·21 (+4·39 to +8·06)	<0·0001
Ecuador	21·01 (21·00–21·02)	28·80 (28·78–28·81)	+3·27 (+0·82 to +5·77)	0·014
Mexico	15·00 (15·00–15·01)	18·35 (18·34–18·35)	+2·69 (+1·79 to +3·59)	0·0001
Peru	10·20 (10·20–10·21)	13·07 (13·06–13·08)	+4·27 (+0·91 to +7·75)	0·018
Puerto Rico	141·97 (141·91–142·04)	178·11 (178·04–178·19)	+2·16 (+1·51 to +2·82)	<0·0001
Uruguay	82·50 (82·45–82·55)	98·80 (98·74–98·86)	+1·84 (+1·26 to +2·42)	<0·0001
Venezuela	53·64 (53·62–53·65)	13·83 (13·82–13·84)	−16·21 (−24·06 to −7·54)	0·0028
**Western Europe**	97·91 (79·52–120·54)	109·52 (88·02–136·27)	+1·13 (+0·62 to +1·64)	<0·0001
Austria	87·68 (87·64–87·71)	106·71 (106·67–106·74)	+1·90 (+1·54 to +2·25)	<0·0001
Belgium	158·82 (158·78–158·86)	174·52 (174·48–174·56)	+1·34 (+0·49 to +2·20)	0·0059
France	99·91 (99·90–99·92)	103·75 (103·74–103·76)	+0·45 (+0·25 to +0·65)	0·0006
Germany	96·09 (96·08–96·10)	121·32 (121·30–121·33)	+2·39 (+2·07 to +2·70)	<0·0001
Luxembourg	93·16 (93·02–93·29)	98·49 (98·36–98·61)	+0·59 (+0·30 to +0·88)	0·0014
Netherlands	81·27 (81·25–81·29)	81·95 (81·93–81·97)	+0·02 (−0·16 to +0·20)	0·83
Switzerland	85·20 (85·16–85·23)	99·89 (99·85–99·92)	+1·63 (+1·25 to +2·01)	<0·0001
**Northern Europe**	87·06 (65·50–115·71)	104·19 (84·03–129·19)	+1·81 (+0·84 to +2·79)	0·0004
Estonia	61·26 (61·19–61·33)	70·83 (70·75–70·90)	+1·41 (+1·11 to +1·71)	<0·0001
Finland	128·65 (128·60–128·70)	140·73 (140·68–140·78)	+1·08 (+0·78 to +1·37)	<0·0001
Ireland	120·78 (120·73–120·84)	135·61 (135·56–135·66)	+0·91 (+0·18 to +1·65)	0·020
Latvia	47·76 (47·71–47·81)	81·95 (81·88–82·02)	+4·32 (+2·48 to +6·20)	0·0005
Lithuania	80·39 (80·34–80·45)	83·79 (83·73–83·84)	+0·13 (−1·66 to +1·96)	0·87
Norway	106·99 (106·94–107·04)	117·60 (117·55–117·65)	+1·11 (+0·88 to +1·34)	<0·0001
Sweden	84·99 (84·96–85·03)	101·61 (101·58–101·65)	+1·93 (+1·53 to +2·32)	<0·0001
United Kingdom	99·29 (99·28–99·31)	125·22 (125·21–125·23)	+2·52 (+2·02 to +3·02)	<0·0001
**Southern Europe**	87·82 (66·97–115·14)	124·17 (99·76–154·56)	+3·52 (+2·76 to +4·29)	<0·0001
Bosnia and Herzegovina	47·03 (46·99–47·06)	84·36 (84·31–84·42)	+5·65 (+4·63 to +6·68)	<0·0001
Croatia	79·59 (79·55–79·63)	110·91 (110·85–110·96)	+3·38 (+3·06 to +3·71)	<0·0001
Greece	87·27 (87·24–87·30)	138·94 (138·90–138·98)	+4·72 (+4·28 to +5·16)	<0·0001
Italy	111·46 (111·45–111·48)	119·30 (119·29–119·32)	+0·58 (+0·24 to +0·92)	0·0039
Portugal	126·31 (126·27–126·34)	168·23 (168·19–168·27)	+3·17 (+2·90 to +3·45)	<0·0001
Serbia	91·61 (91·58–91·65)	163·54 (163·49–163·59)	+6·03 (+5·05 to +7·02)	<0·0001
Slovenia	70·26 (70·20–70·32)	89·74 (89·67–89·80)	+2·35 (+1·98 to +2·72)	<0·0001
Spain	119·50 (119·48–119·51)	147·61 (147·59–147·62)	+2·25 (+2·05 to +2·45)	<0·0001
**Eastern Europe**	60·36 (38·35–95·00)	77·35 (53·49–111·86)	+2·51 (+1·65 to +3·38)	<0·0001
Belarus	30·91 (30·89–30·93)	43·29 (43·26–43·31)	+3·71 (+2·87 to +4·56)	<0·0001
Bulgaria	66·85 (66·82–66·88)	87·76 (87·73–87·80)	+2·55 (+2·00 to +3·10)	<0·0001
Czech Republic	80·90 (80·87–80·93)	108·04 (108·01–108·07)	+2·61 (+1·91 to +3·31)	<0·0001
Hungary	112·23 (112·20–112·27)	113·43 (113·39–113·46)	−0·14 (−0·59 to +0·31)	0·50
Poland	80·95 (80·94–80·97)	104·58 (104·56–104·59)	+2·31 (+1·68 to +2·94)	<0·0001
Romania	73·65 (73·63–73·67)	84·68 (84·66–84·70)	+1·18 (+0·57 to +1·81)	0·0019
Russia	22·40 (22·40–22·41)	34·92 (34·92–34·93)	+4·03 (+2·97 to +5·09)	<0·0001
Slovakia	70·33 (70·29–70·36)	89·04 (89·00–89·08)	+1·81 (+1·07 to +2·55)	0·0004
**Oceania**	98·88 (65·27–149·80)	107·30 (49·49–232·63)	+0·82 (+0·29 to +1·35)	0·0043
Australia	102·16 (102·14–102·19)	114·04 (114·02–114·06)	+1·11 (+0·68 to +1·53)	0·0002
New Zealand	95·70 (95·65–95·75)	100·96 (100·92–101·01)	+0·46 (+0·09 to +0·83)	0·021
**Eastern Asia**	35·45 (9·32–134·92)	48·13 (15·20–152·44)	+3·10 (+2·27 to +3·95)	<0·0001
China	5·36 (5·36–5·36)	9·44 (9·44–9·44)	+6·12 (+5·50 to +6·74)	<0·0001
Hong Kong	48·58 (48·55–48·61)	59·39 (59·36–59·42)	+2·28 (+1·81 to +2·74)	<0·0001
Japan	79·51 (79·50–79·52)	89·37 (89·36–89·38)	+1·51 (+1·16 to +1·87)	<0·0001
South Korea	47·46 (47·45–47·47)	61·35 (61·34–61·36)	+2·57 (+2·23 to +2·91)	<0·0001
Taiwan	57·03 (57·02–57·05)	84·01 (83·99–84·02)	+3·83 (+3·63 to +4·02)	<0·0001
**Central Asia**	16·72 (16·71–16·73)	23·02 (23·01–23·03)	+3·33 (+2·44 to +4·22)	<0·0001
Kazakhstan	16·72 (16·71–16·73)	23·02 (23·01–23·03)	+3·33 (+2·41 to +4·25)	<0·0001
**Western Asia**	14·29 (4·18–48·89)	22·08 (7·37–66·16)	+4·70 (+0·58 to +8·99)	0·026
Jordan	9·57 (9·56–9·58)	13·42 (13·41–13·44)	+2·59 (+0·21 to +5·04)	0·036
Kuwait	2·29 (2·28–2·30)	4·34 (4·33–4·35)	+9·99 (+4·31 to +15·98)	0·0028
Lebanon	46·47 (46·44–46·51)	25·39 (25·37–25·41)	+0·20 (−4·98 to +5·66)	0·93
Saudi Arabia	16·17 (16·16–16·18)	57·12 (57·10–57·13)	+11·45 (+4·45 to +18·92)	0·0044
Türkiye	54·41 (54·40–54·41)	78·09 (78·08–78·10)	+3·68 (+3·09 to +4·28)	<0·0001
United Arab Emirates	9·48 (9·47–9·49)	17·58 (17·57–17·59)	+7·12 (+4·41 to +9·90)	0·0002
**South-eastern Asia**	8·17 (2·43–27·41)	13·56 (4·61–39·92)	+5·20 (+3·41 to +7·03)	<0·0001
Indonesia	1·92 (1·92–1·93)	3·75 (3·75–3·75)	+7·41 (+2·66 to +12·37)	0·0059
Malaysia	17·11 (17·10–17·12)	22·17 (22·16–22·18)	+1·39 (−0·33 to +3·15)	0·10
Philippines	3·42 (3·42–3·42)	6·73 (6·73–6·73)	+6·60 (+5·89 to +7·31)	<0·0001
Singapore	26·14 (26·12–26·17)	35·16 (35·13–35·18)	+2·89 (+2·12 to +3·67)	<0·0001
Thailand	26·09 (26·08–26·09)	46·55 (46·54–46·56)	+5·59 (+4·56 to +6·63)	<0·0001
Vietnam	3·85 (3·85–3·86)	6·80 (6·80–6·80)	+7·50 (+4·90 to +10·17)	0·0001
**Southern Asia**	14·18 (7·53–26·70)	20·59 (12·67–33·47)	+3·80 (+1·52 to +6·14)	0·0016
Bangladesh	10·03 (10·02–10·03)	21·97 (21·96–21·97)	+8·26 (+6·32 to +10·24)	<0·0001
India	23·61 (23·61–23·61)	30·39 (30·39–30·39)	+2·80 (+2·51 to +3·09)	<0·0001
Pakistan	10·68 (10·67–10·68)	18·18 (18·18–18·19)	+5·21 (+4·74 to +5·67)	<0·0001
Sri Lanka	15·98 (15·97–15·99)	14·82 (14·81–14·83)	−0·21 (−1·97 to +1·59)	0·80
**Northern Africa**	30·75 (11·57–81·69)	41·74 (18·57–93·80)	+3·10 (+0·79 to +5·47)	0·0096
Algeria	42·30 (42·29–42·31)	48·04 (48·03–48·05)	+2·23 (+0·96 to +3·51)	0·0031
Egypt	21·11 (21·10–21·11)	50·96 (50·96–50·97)	+10·58 (+8·56 to +12·65)	<0·0001
Morocco	16·26 (16·25–16·27)	19·83 (19·82–19·84)	+1·98 (+1·49 to +2·48)	<0·0001
Tunisia	61·57 (61·55–61·59)	62·52 (62·50–62·54)	−0·04 (−1·14 to +1·07)	0·93
**Southern Africa**	59·59 (59·58–59·60)	62·79 (62·78–62·80)	+0·75 (−2·73 to +4·35)	0·64
South Africa	59·59 (59·58–59·60)	62·79 (62·78–62·80)	+0·79 (−1·99 to +3·65)	0·54

CI = Confidence interval; DDD/TID = Defined Daily Dose per 10,000 inhabitants per day.

a Worldwide and regional estimates with 95% CI were calculated by pooling the estimates using meta-analysis (random-effects model).

b The average annual percentage change is calculated using a linear regression model, with log-transformed consumption in DDD/TID as the dependent variable and year as the independent variable. The average annual change was expressed as average annual percentage change, calculated by [exp(the coefficient of the year variable) − 1] × 100%. The multinational and regional trend changes were estimated using linear mixed models, controlling for within-country correlations and assuming the correlations between years were autocorrelated.

Annual average percentage changes of ASM consumption rate were similar across country income levels. The average annual increase in lower-middle income countries/regions (n = 12) was +3·13% (+1·10% to +5·20%) from 15·63 DDD/TID (8·75–27·91) in 2012 to 21·26 DDD/TID (13·43–33·65) in 2022, followed by upper-middle countries/regions (n = 20) with an average annual increase of +2·73% (+0·95% to +4·55%) from 24·34 DDD/TID (15·19–38·97) in 2012 to 31·87 DDD/TID (20·12–50·47) in 2022, and high-income countries/regions (n = 41) with an average annual increase of +2·35% (+1·66% to +3·06%) from 70·01 DDD/TID (54·54–89·87) in 2012 to 88·36 DDD/TID (71·69–108·90) in 2022 (Table 2).

**Table 2:** Annual pooled antiseizure medication consumption rate and average annual percentage change from 2012 to 2022 by country income level.

Income level	High (n = 41)	Upper-middle (n = 20)	Lower-middle (n = 12)
Average annual percentage change^a^ (%, 95% CI)	+2·35 (+1·66 to +3·06)	+2·73 (+0·95 to +4·55)	+3·13 (+1·10 to +5·20)
**Year**	**Pooled consumption rate (defined daily dose per 10,000 inhabitants per day)**		
2012	70·01 (54·54–89·87)	24·34 (15·19–38·97)	15·63 (8·75–27·91)
2013	72·75 (56·49–93·70)	25·34 (15·96–40·24)	16·11 (9·46–27·45)
2014	75·72 (59·10–97·02)	25·62 (15·85–41·41)	16·01 (9·28–27·62)
2015	77·00 (60·03–98·76)	26·82 (16·98–42·37)	16·86 (9·99–28·47)
2016	77·36 (59·78–100·12)	25·79 (16·33–40·71)	17·96 (10·71–30·13)
2017	80·82 (63·88–102·25)	26·72 (17·02–41·95)	18·52 (11·13–30·80)
2018	81·49 (64·46–103·03)	27·86 (17·70–43·86)	19·92 (12·14–32·68)
2019	83·45 (66·30–105·04)	28·60 (18·04–45·33)	20·91 (12·71–34·40)
2020	86·48 (69·60–107·46)	31·11 (19·71–49·13)	22·25 (13·44–36·85)
2021	88·10 (72·00–107·80)	30·78 (19·55–48·47)	21·58 (13·13–35·49)
2022	88·36 (71·69–108·90)	31·87 (20·12–50·47)	21·26 (13·43–33·65)

CI = Confidence interval.

a The average annual change is calculated using a linear regression model, with log-transformed consumption rate in DDD/TID as the dependent variable and year as the independent variable. The average annual change was expressed as average annual percentage change, calculated by [exp(the coefficient of the year variable) − 1] × 100%.

**Fig. 3:**
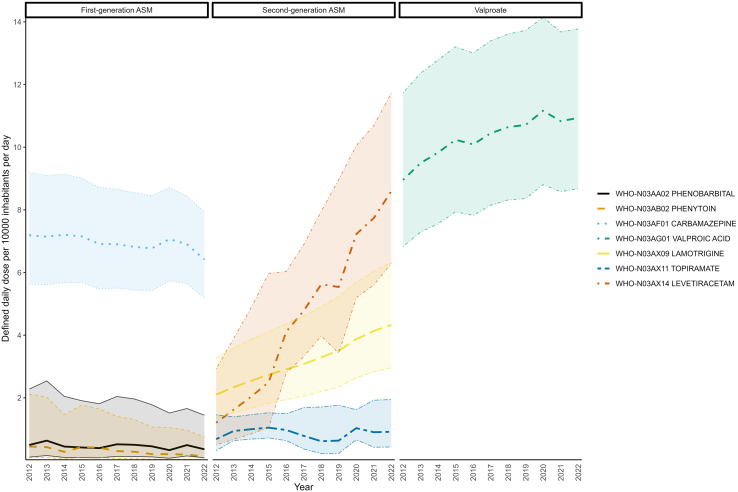
**Multinational consumption of seven selected antiseizure medications from 2012 to 2022.** Different colours represent the antiseizure medications included in this figure, while the shaded areas indicate the 95% CI of defined daily doses per 10,000 inhabitants per day from 2012 to 2022. ASM = Antiseizure medications.

The average annual changes for different ASMs from 2012 to 2022 multi-nationally are presented in Supplementary Table S3. Despite an overall increase in the ASM consumption rate of, shifting consumption patterns between different ASMs were observed during the study period. In 2022, the top five most consumed ASMs were valproate, levetiracetam, carbamazepine, lamotrigine and clonazepam (Supplementary Table S4). Multi-nationally, use of older generation ASMs notably phenobarbital (−2·85%, −9·50% to +4·29%), phenytoin (−11·19%, −17·58% to −4·30%), and carbamazepine (−1·09%, −1·95% to −0·23%), declined over the study period (Fig. 3). In contrast, consumption of newer ASMs rose sharply. Among the top ten most consumed ASMs, levetiracetam showed an average annual increase in consumption of +21·72% (+13·86% to +30·11%) from 1·21 DDD/TID (0·50–2·92) in 2012 to 8·59 DDD/TID (6·30–11·72) in 2022, and lamotrigine which showed an average annual increase in consumption of +7·48% (+6·34% to +8·63%) from 2·10 DDD/TID (1·35–3·26) in 2012 to 4·32 DDD/TID (2·96–6·32) in 2022 (Supplementary Table S3).

Throughout the study period, valproate remained the most consumed ASM multi-nationally with an average annual percentage change in consumption of +2·02% (+1·12% to +2·92%) from 8·95 DDD/TID (6·83–11·74) DDD/TID in 2012 to 10·93 DDD/TID (8·68–13·77) DDD/TID in 2022 (Supplementary Tables S4 and S5 and Supplementary Figure S2). Amongst the 73 countries/regions studied, valproate consumption rate increased in 43 countries, remained stable in 14 countries, and decreased in 16 countries (Supplementary Figure S3). In 2022, Belgium had the highest consumption rate of valproate (64·80 DDD/TID, 64·78–64·83), followed by Poland (34·67 DDD/TID, 34·66–34·68) and Finland (33·13 DDD/TID, 33·11–33·16). Between 2012 and 2022, excluding Venezuela, Ireland (−5·35%, −7·41% to −3·24%), Luxembourg (−4·23%, −4·47% to −3·98%), and Netherlands (−2·67%, −3·11% to −2·23%) recorded the biggest drop in valproate consumption (Supplementary Table S5, Supplementary Figures S3 and S4). When stratified by country income level, valproate consumption from 2012 to 2022 remained stable in high-income countries, with an average annual percentage change of +0·86% (−0·07% to +1·79%), and increased in upper-middle income countries and lower-middle income countries with an average annual percentage change of +3·10% (+0·95% to +5·29%) and +4·24% (+1·73% to +6·81%), respectively (Supplementary Table S6).

In the sensitivity analysis where the consumption rates were calculated as DDD/HED, the results were consistent with the main analyses with an increase in multinational consumption rate +2·88% (+2·09% to +3·67%), from 57·63 DDD/HED (44·62–74·44) in 2012 to 74·40 DDD/HED (58·75–94·22) in 2021 (Supplementary Table S7 and S8). Valproate, levetiracetam, carbamazepine, lamotrigine, and clonazepam continued to exhibit the highest consumption rates from 2012 to 2021, showing similar consumption trends across geographical regions and income levels compared with the main analysis (Supplementary Table S9).

## Discussion

Between 2012 and 2022, our study showed an increasing consumption rate of ASMs in 73 countries/regions, across country income levels and geographical regions, with an average annual growth of 2·58%. Venezuela was the only country that recorded a significant decline in ASM consumption rate between 2012 and 2022, likely reflecting its economic downturn driven by international sanctions and reduced health expenditure which began in the early 2010s.^38^^,^^39^ Newer generation ASMs drove most of the overall growth, with levetiracetam and lamotrigine rising at average annual rates of 21·72% and 7·48%, respectively. The multinational consumption rate of valproate increased and it remained the most consumed ASM, with varied trends across countries and income groups. In contrast, the consumption of other older agents such as phenobarbital, phenytoin and carbamazepine declined. Absolute consumption rate of ASMs remained highest in high-income countries.

The findings in our study have several global policy and clinical implications. The increase in ASM consumption likely reflects a combination of expanding indications to include conditions other than epilepsy, greater access to medications, and evolving clinical practice.^40^^,^^41^ In 2009, the WHO estimated that up to 75% of people with epilepsy in low-income countries do not receive treatment, despite the availability of inexpensive and effective medications.^42^ WHO's intersectoral global action plan on epilepsy and other neurological disorders 2022–2031^43^ emphasises the need for appropriate, transparent and sustainable use of essential medicines for the treatment of neurological disorders including epilepsy. While the increase in ASM consumption in lower income countries/regions marked an encouraging sign for increased access to antiseizure medication, disparities in absolute consumption persist. Our study showed that, in 2012, ASM consumption rates in high-income countries were approximately 4·5 times those in lower-middle-income countries, and by 2022 this gap had only marginally narrowed to 4·15 times. This phenomenon remains the same in our sensitivity analysis where we accounted for the prevalence of epilepsy in different countries. These disparities align with previous work demonstrating unequal access to antiseizure medication globally.^44^ Echoing United Nations Sustainable Development Goal 3·8—“Achieve universal health coverage, including financial risk protection, access to quality essential health-care services, and access to safe, effective, quality and affordable essential medicines and vaccines for all”—we urge continued efforts to broaden access to different ASMs, especially those on the WHO List of Essential Medicines, and in lower-income countries.^45^

Despite the overall rise in ASM consumption, we observed a decline in phenobarbital, phenytoin, and carbamazepine, which may be attributable to their less favourable safety profiles, with various drug-specific concerns.^46^^,^^47^ The receding of these older agents may benefit the rise of newer agents such as levetiracetam and lamotrigine, which have demonstrated a safer profile and are easier to use in comparison.^48^^,^^49^ A marked increase of levetiracetam consumption in upper-middle and lower-middle countries suggests broadening access to first-line newer therapies outside of high-income countries. The expiry of its patent shortly before the start of the study period also catalysed the entry of low-cost generics, further accelerating use.^50^ With the inclusion of lamotrigine and levetiracetam into the WHO List of Essential Medicines in 2017 and 2023 respectively,^51^ their increasing consumption trend may persist. Encouragingly, a growing number of countries are now incorporating these comparatively safer ASMs into their own national essential medicines lists.^52^ Nevertheless, sustained progress will depend on complementary measures, including timely revision of national standard treatment guidelines and essential medicines lists, to ensure that national policies consistently align with the latest evidence and guidance around essential antiseizure medicines. Meanwhile, newly marketed third generation medications such as lacosamide, perampanel, or brivaracetam are posting very large relative increases, although their absolute consumption rates remain modest, and this growth is concentrated in high-income countries that can more readily afford costlier innovations. Given their recent introduction, future studies evaluating their effectiveness and safety in real-world clinical practice are needed.

Valproate remained the most-consumed ASM throughout the study period, even though decades of evidence have highlighted its teratogenic and neurodevelopmental hazards.^53^ Amongst the 73 countries/regions included, 16 of them recorded a significant decrease in consumption rates while global consumption moderately increased, driven mainly by lower- and upper-middle-income countries. The affordability and broad-spectrum efficacy of valproate^3^^,^^51^ may help to explain its continued rise where newer ASMs remain prohibitively expensive, especially in lower-income settings. Small declines in Oceania, Western Europe and Northern America, consistent with prior country-level analyses in high-income settings,^54^, ^55^, ^56^, ^57^ may suggest that valproate is increasingly avoided as a first- or second-line antiseizure therapy and reflect changes in prescribing practice following safety warnings, prescribing restrictions, and pregnancy prevention programmes introduced by the EMA, MHRA, and FDA.^4^^,^^8^^,^^9^^,^^11^^,^^14^ International patient-level studies examining compliance with prescribing recommendations and safety guidance issued by health authorities are warranted.

However, the exact definitions of these prescribing restrictions vary by jurisdiction. For instance, the UK MHRA recommends that valproate should not be started in any patient (both women and men) under 55 years of age without approval from two specialists.^58^ In contrast, the EMA requires only women of childbearing potential, without a numeric age threshold, use effective contraception throughout valproate treatment and for one month after discontinuation; additionally, in 2024, valproate therapy in male patients is recommended to be started and supervised by a specialist.^9^^,^^13^ The US FDA disseminates risk information via labelling and educational tools without compulsory pregnancy prevention programmes.^14^ Throughout the study period, warnings and restrictions on valproate were introduced at different time points. Although these measures apply only to girls and women of childbearing potential, the lack of standardisation may contribute to prescriber confusion and inconsistent application of risk minimisation measures,^59^ leading to a steady consumption trajectory of valproate from high income countries, mirroring the findings of some previous reports.^17^^,^^18^

Regarding paternal valproate exposure, the MHRA's and EMA's additional measures were issued in early 2024, which was outside of the study period (2012–2022), and therefore could not have influenced the trends reported in this study. Recent studies evaluating neurodevelopmental outcomes in the offspring of men exposed to valproate have also yielded conflicting results.^60^, ^61^, ^62^, ^63^ The recent introduction of structured pregnancy prevention programmes in several jurisdictions^4^^,^^9^ and precautionary warning of potential risk of neurodevelopmental disorders in children born to men treated with valproate,^12^^,^^13^^,^^63^ warrants follow-up study of valproate and consumption of alternatives which will allow us to monitor and quantify how these measures influence valproate prescribing in the years ahead.

Nevertheless, valproate has also been shown to be one of the most clinically and cost-effective treatment options, for instance, in idiopathic generalised epilepsy when compared with levetiracetam.^64^ In parallel, we cannot ignore that in some clinical settings, particularly where diagnostic uncertainty persists or access to newer agents is limited, valproate may be the most effective, and in some circumstances the safest option given the immediate hazards and potential life-threatening nature of uncontrolled seizures. The choice of antiseizure treatment should therefore be patient-centred and guided by a rigorous risk-benefit assessment.

The MIDAS database offers a platform for global comparison of ASM consumption across different geographical regions and income levels. Previous studies mainly focussed on a single country, a specific patient group or excluded lower income countries.^18^, ^19^, ^20^, ^21^^,^^54^, ^55^, ^56^, ^57^ Our study is the first study to report on the international trends in the consumption of ASMs, including high-income, upper- and lower-middle-income countries/regions. The use of DDD/TID and DDD/HED as a study measures offer us insights on the absolute consumption of ASMs and the consumption changed in accordance with the prevalence of epilepsy in different countries/regions. However, our study has limitations. First, the MIDAS database is a sales database; information on patient's age, gender, duration of prescription, medical diagnosis and concomitant medications is not available. Thus, our design quantifies country-level consumption and does not permit a detailed characterisation of prescribing behaviours, clinical decision-making, or trends within specific patient subgroups. However, our sensitivity analysis taking into account of the epilepsy population does not change the trend that was observed in the main analysis. Further investigation into age-standardised consumption rates of antiseizure medications is warranted. Second, although 77% of the world population were included in our study, the findings do not necessarily apply to the countries that are not included in the dataset, including many countries in the African region. Third, the database provides sales figures related only to legitimate means of distribution. Illicit sales of drugs are not captured and consequently the data presented in the study may not reflect the pattern of illegal consumption in the countries analysed. Finally, as this is a descriptive study with potential for unexplored variables, we cannot conclude a causal link between any factors and our observed ASM consumption trends.

In conclusion, between 2012 and 2022, global consumption of ASMs, across geographical regions and income levels increased, driven mainly by newer-generation medications such as levetiracetam and lamotrigine. Nevertheless, valproate remains the most-widely used medication, with consumption patterns varying considerably across regions. These findings underscore potential disparities in ASM access, particularly in lower- and upper-middle-income countries. Ongoing global efforts are essential to align prescribing practices with the latest safety warnings and guidelines, in order to optimise patient outcomes. Such measures are critical to ensuring both equitable and responsible use of ASMs.

## Contributors

AYLC, ASCY, KKCM, and ICKW had direct access to the aggregate analysis data in the study and verified the underlying data reported in the manuscript.

ASCY conducted the literature search.

AYLC prepared the figures.

ICKW and KKCM conceived the study and provided supervision.

AYLC and ASCY curated the data and performed the formal analyses.

ICKW, KKCM, AYLC, and ASCY were responsible for the study design.

AYLC and ASCY drafted the original manuscript.

ICKW acquired the funding of this study.

All authors contributed to data interpretation, and participated in reviewing and editing the manuscript.

## Data sharing statement

The underlying MIDAS data were provided by IQVIA under licence. The terms of our agreement do not permit disclosure, sublicensing, or sharing of IQVIA-MIDAS data. IQVIA will honour legitimate requests for MIDAS data from qualified researchers. Please contact IQVIA to seek approval for data access; a licence fee may be applied.

## Editor note

The *Lancet* Group takes a neutral position with respect to territorial claims in published maps and institutional affiliations. The authors alone are responsible for the views expressed in this article and they do not necessarily represent the views, decisions or policies of the institutions with which they are affiliated. This is an Open Access article published under the CC BY 3.0 IGO license which permits unrestricted use, distribution, and reproduction in any medium, provided the original work is properly cited. In any use of this article, there should be no suggestion that WHO endorses any specific organisation, products or services. The use of the WHO logo is not permitted. This notice should be preserved along with the article’s original URL.

## Declaration of interests

All authors have completed the ICMJE uniform disclosure form at www.icmje.org/coi_disclosure.pdf and declare:

AYLC reports grant from the AIR@innoHK programme of the Hong Kong Innovation and Technology Commission; ASCY reports grant from the University College London Hospitals NIHR Biomedical Research Centre, College of Mental Health Pharmacy, and UK Turing Scheme; WCYL reports grant from AIR@InnoHK administered by Innovation and Technology Commission, outside the submitted work. JHC has acted as an investigator for studies with Jazz/GW Pharmaceuticals, Stoke Therapeutics, UCB/Zogenix, Ultragenyx, Encoded, and Vitaflo; has been a speaker and has served on advisory boards for Biocodex, Jazz Pharmaceuticals, Nutricia, Stoke Therapeutics, and UCB (all remuneration has been paid to her department); holds an endowed chair at the University College of London Great Ormond Street Institute of Child Health; has received grants from the National Institute for Health and Care Research (NIHR), the Engineering and Physical Sciences Research Council (EPSRC), the Great Ormond Street Hospital for Children (GOSH) Charity, LifeArc, and Epilepsy Research UK; and her research is supported by the NIHR Great Ormond Street Hospital Biomedical Research Centre. MCW has consulted for Angelini Pharma, EpilepsyGtX, and Seer and has received honoraria from Angelini Pharma, Bioquest, Eisai, and UCB pharma, received payment for expert testimony for Pfizer, act as the Chair of trustees of Epilepsy Research Institute, and owns shares in EpilepsyGtx; MCW is also associated with the following patents: WO2018229254A1, EP3116508A1, US10301263B2, EP2642990B1, CA3064329A1, and WO2023152318A1. ATFH is the Vice President (Western Pacific) of International Bureau for Epilepsy and act as an advisor of Hong Kong Epilepsy Association. KKCM reports grants from the CW Maplethorpe Fellowship, the European Union Horizon 2020, the UK National Institute of Health Research and the Hong Kong Research Grant Council, Hong Kong Innovation and Technology Commission, and reports personal fees from IQVIA, unrelated to the submitted work. ICKW received payment for expert testimony for Appeal Court in Hong Kong; ICKW serves on advisory committees for Member of Hong Kong Pharmacy and Poisons Board, as a member of the Expert Committee on Clinical Events Assessment Following COVID-19 Immunisation in Hong Kong, as a member of the Advisory Panel on COVID-19 Vaccines of the Hong Kong Government, as the non-executive director of Jacobson Pharma Corp. Ltd. In Hong Kong, as the founder and director of Therakind Limited (UK), Advance Data Analytics for Medical Science (ADAMS) Limited (HK) and OCUS Innovation Limited (HK, Ireland, and UK); no other relationships or activities that could appear to have influenced the submitted work. YH, FMCB, NI, and NC declare no competing interests.
