# Supplementary material for: Antiseizure medications consumption in 73 countries and regions from 2012 to 2022: a longitudinal trend study
Source: eClinicalMedicine. 2025 Oct 9;89:103558. doi: 10.1016/j.eclinm.2025.103558 (PMC12547185; doi:10.1016/j.eclinm.2025.103558)
Supplement: Supplementary Tables and Figures [file mmc1.pdf]

Antiseizure medications consumption in 73 countries and regions from 2012 to 2022: a longitudinal trend study

Supplementary materials

Supplementary Table 1. Defined daily dose of different antiseizure medications according to the World Health Organisation Anatomical Therapeutic Chemical Classification System.

ATC code	Antiseizure medication	Antiseizure medications classification	Defined daily dose	Unit	Year since sales is recorded in the study
N03AA01	Methylphenobarbital	Barbiturates and derivatives	1·8	g	2012
N03AA02	Phenobarbital	Barbiturates and derivatives	0·1	g	2012
N03AA03	Primidone	Barbiturates and derivatives	1·25	g	2012
N03AB01	Ethotoin	Hydantoin derivatives	2·5	g	2012
N03AB02	Phenytoin	Hydantoin derivatives	0·3	g	2012
N03AB05	Fosphenytoin	Hydantoin derivatives	0·45	g	2012
N03AC02	Trimethadione	Oxazolidine derivatives	1·5	g	2012
N03AD01	Ethosuximide	Succinimide derivatives	1·25	g	2012
N03AD03	Mesuximide	Succinimide derivatives	0·9	g	2012
N03AE01	Clonazepam	Benzodiazepine derivatives	8	mg	2012
N03AF01	Carbamazepine	Carboxamide derivatives	1	g	2012
N03AF02	Oxcarbazepine	Carboxamide derivatives	1	g	2012
N03AF03	Rufinamide	Carboxamide derivatives	1·4	g	2012
N03AF04	Eslicarbazepine	Carboxamide derivatives	0·8	g	2012
N03AG01	Valproic acid (valproate)	Fatty acid derivatives	1·5	g	2012
N03AG02	Valpromide	Fatty acid derivatives	1·5	g	2012
N03AG03	Aminobutyric acid	Fatty acid derivatives	1	g	2012
N03AG04	Vigabatrin	Fatty acid derivatives	2	g	2012
N03AG06	Tiagabine	Fatty acid derivatives	30	mg	2012
N03AX03	Sultiame	Other antiepileptics	0·4	g	2012
N03AX09	Lamotrigine	Other antiepileptics	0·3	g	2012
N03AX10	Felbamate	Other antiepileptics	2·4	g	2012
N03AX11	Topiramate	Other antiepileptics	0·3	g	2012
N03AX14	Levetiracetam	Other antiepileptics	1·5	g	2012
N03AX15	Zonisamide	Other antiepileptics	0·2	g	2012
N03AX17	Stiripentol	Other antiepileptics	1	g	2012
N03AX18	Lacosamide	Other antiepileptics	0·3	g	2012
N03AX21	Retigabine	Other antiepileptics	0·9	g	2012
N03AX22	Perampanel	Other antiepileptics	8	mg	2012
N03AX23	Brivaracetam	Other antiepileptics	0·1	g	2016
N03AX24	Cannabidiol	Other antiepileptics	0·7	g	2018
N03AX25	Cenobamate	Other antiepileptics	0·2	g	2020
N03AX26	Fenfluramine	Other antiepileptics	8	mg	2021

ATC = Anatomical therapeutic chemical.

Supplementary Table 2. Geographical regions, income classifications of countries/ regions and their market coverage in MIDAS.

Country/ Region	Geographical regions according to World Bank	Geographical regions according to WHO	Income level	Sectors covered	Market coverage (%)
Canada	Northern America	Region of the Americas	HIC	Hospital and Retail	100
United States	Northern America	Region of the Americas	HIC	Hospital and Retail	89
Argentina	Central and South America and the Caribbean	Region of the Americas	UMIC	Retail	73
Brazil	Central and South America and the Caribbean	Region of the Americas	UMIC	Hospital and Retail	97
Chile	Central and South America and the Caribbean	Region of the Americas	HIC	Retail	71
Colombia	Central and South America and the Caribbean	Region of the Americas	UMIC	Retail	71
Dominican Republic	Central and South America and the Caribbean	Region of the Americas	UMIC	Retail	78
Ecuador	Central and South America and the Caribbean	Region of the Americas	UMIC	Retail	80
Mexico	Central and South America and the Caribbean	Region of the Americas	UMIC	Hospital and Retail	100
Peru	Central and South America and the Caribbean	Region of the Americas	UMIC	Retail	67
Puerto Rico	Central and South America and the Caribbean	NA	HIC	Hospital and Retail	93
Uruguay	Central and South America and the Caribbean	Region of the Americas	HIC	Retail and others	71
Venezuela	Central and South America and the Caribbean	Region of the Americas	UMIC	Retail	78
Austria	Western Europe	European Region	HIC	Hospital and Retail	100
Belgium	Western Europe	European Region	HIC	Hospital and Retail	99
France	Western Europe	European Region	HIC	Hospital and Retail	100
Germany	Western Europe	European Region	HIC	Hospital and Retail	100

Luxembourg	Western Europe	European Region	HIC	Retail	98
Netherlands	Western Europe	European Region	HIC	Hospital and Retail	91
Switzerland	Western Europe	European Region	HIC	Hospital and Retail	100
Estonia	Northern Europe	European Region	HIC	Retail	88
Finland	Northern Europe	European Region	HIC	Hospital and Retail	100
Ireland	Northern Europe	European Region	HIC	Hospital and Retail	100
Latvia	Northern Europe	European Region	HIC	Hospital and Retail	100
Lithuania	Northern Europe	European Region	HIC	Hospital and Retail	99
Norway	Northern Europe	European Region	HIC	Hospital and Retail	100
Sweden	Northern Europe	European Region	HIC	Hospital and Retail	100
United Kingdom	Northern Europe	European Region	HIC	Hospital and Retail	89
Bosnia and Herzegovina	Southern Europe	European Region	UMIC	Hospital and Retail	95
Croatia	Southern Europe	European Region	HIC	Hospital and Retail	98
Greece	Southern Europe	European Region	HIC	Retail	60
Italy	Southern Europe	European Region	HIC	Hospital and Retail	99
Portugal	Southern Europe	European Region	HIC	Hospital and Retail	100
Serbia	Southern Europe	European Region	UMIC	Hospital and Retail	93
Slovenia	Southern Europe	European Region	HIC	Hospital and Retail	98
Spain	Southern Europe	European Region	HIC	Hospital and Retail	99

Belarus	Eastern Europe	European Region	UMIC	Hospital and Retail	100
Bulgaria	Eastern Europe	European Region	UMIC	Hospital and Retail	98
Czech Republic	Eastern Europe	European Region	HIC	Hospital and Retail	95
Hungary	Eastern Europe	European Region	HIC	Hospital and Retail	100
Poland	Eastern Europe	European Region	HIC	Hospital and Retail	100
Romania	Eastern Europe	European Region	HIC	Hospital and Retail	100
Russia	Eastern Europe	European Region	UMIC	Hospital and Retail	97
Slovakia	Eastern Europe	European Region	HIC	Hospital and Retail	97
Australia	Oceania	Western Pacific Region	HIC	Hospital and Retail	90
New Zealand	Oceania	Western Pacific Region	HIC	Hospital and Retail	97
China	Eastern Asia	Western Pacific Region	UMIC	Hospital and Retail	72
Hong Kong	Eastern Asia	NA	HIC	Hospital and Retail	91
Japan	Eastern Asia	Western Pacific Region	HIC	Hospital and Retail	100
South Korea	Eastern Asia	Western Pacific Region	HIC	Hospital and Retail	99
Taiwan	Eastern Asia	NA	HIC	Hospital and Retail	97
Kazakhstan	Central Asia	European Region	UMIC	Hospital and Retail	100
Jordan	Western Asia	Eastern Mediterranean Region	LMIC	Retail	71
Kuwait	Western Asia	Eastern Mediterranean Region	HIC	Hospital and Retail	35
Lebanon	Western Asia	Eastern Mediterranean Region	LMIC	Retail	77

Saudi Arabia	Western Asia	Eastern Mediterranean Region	HIC	Hospital and Retail	85
Türkiye	Western Asia	European Region	UMIC	Hospital and Retail	100
United Arab Emirates	Western Asia	Eastern Mediterranean Region	HIC	Hospital and Retail	45
Indonesia	South-eastern Asia	South-East Asia Region	UMIC	Hospital and Retail	60
Malaysia	South-eastern Asia	Western Pacific Region	UMIC	Hospital and Retail	82
Philippines	South-eastern Asia	Western Pacific Region	LMIC	Hospital and Retail	100
Singapore	South-eastern Asia	Western Pacific Region	HIC	Hospital and Retail	87
Thailand	South-eastern Asia	South-East Asia Region	UMIC	Hospital and Retail	93
Vietnam	South-eastern Asia	Western Pacific Region	LMIC	Hospital and Retail	84
Bangladesh	Southern Asia	South-East Asia Region	LMIC	Retail	85
India	Southern Asia	South-East Asia Region	LMIC	Hospital and Retail	95
Pakistan	Southern Asia	Eastern Mediterranean Region	LMIC	Hospital and Retail	85
Sri Lanka	Southern Asia	South-East Asia Region	LMIC	Retail	58
Algeria	Northern Africa	African Region	LMIC	Retail	80
Egypt	Northern Africa	Eastern Mediterranean Region	LMIC	Retail	75
Morocco	Northern Africa	Eastern Mediterranean Region	LMIC	Retail	88
Tunisia	Northern Africa	Eastern Mediterranean Region	LMIC	Hospital and Retail	100
South Africa	Southern Africa	African Region	UMIC	Hospital and Retail	100

*Hong Kong, Puerto Rico and Taiwan do not have a region assigned by WHO.

HIC = High-income country; UMIC = Upper-middle-income country; LMIC = Lower-middle-income country; MIDAS = Multinational Integrated Data Analysis System; WHO = World Health Organisation; NA = Not applicable.

Supplementary Table 3. Multinational consumption rates of individual antiseizure medications in 2012 and 2022, and their average annual percentage change.

Antiseizure medication	DDD/TID in 2012 (95%CI)[a,c]	DDD/TID in 2022 (95%CI)[a,c]	Average annual percentage change (%, 95%CI)[b]	P value
Methylphenobarbital	$9{\cdot}00 \times 10^{-11}$ ($1{\cdot}90 \times 10^{-11}$ to $4{\cdot}26 \times 10^{-10}$)	$5{\cdot}78 \times 10^{-11}$ ($1{\cdot}90 \times 10^{-11}$ to $4{\cdot}26 \times 10^{-10}$)	-3·86 (-6·37 to -1·28)	0·0036
Phenobarbital	$4{\cdot}95 \times 10^{-1}$ ($1{\cdot}07 \times 10^{-1}$ to $2{\cdot}28 \times 10^{0}$)	$3{\cdot}58 \times 10^{-1}$ ($8{\cdot}84 \times 10^{-2}$ to $1{\cdot}45 \times 10^{0}$)	-2·85 (-9·50 to +4·29)	0·42
Primidone	$7{\cdot}27 \times 10^{-6}$ ($4{\cdot}60 \times 10^{-7}$ to $1{\cdot}15 \times 10^{-4}$)	$8{\cdot}23 \times 10^{-6}$ ($5{\cdot}24 \times 10^{-7}$ to $1{\cdot}29 \times 10^{-4}$)	+0·22 (-9·48 to +10·95)	0·97
Ethotoin	$3{\cdot}17 \times 10^{-11}$ ($1{\cdot}16 \times 10^{-11}$ to $8{\cdot}64 \times 10^{-11}$)	$1{\cdot}66 \times 10^{-11}$ ($8{\cdot}76 \times 10^{-12}$ to $3{\cdot}14 \times 10^{-11}$)	-6·26 (-11·69 to -0·51)	0·033
Phenytoin	$4{\cdot}47 \times 10^{-1}$ ($9{\cdot}40 \times 10^{-2}$ to $2{\cdot}12 \times 10^{0}$)	$1{\cdot}30 \times 10^{-1}$ ($2{\cdot}28 \times 10^{-2}$ to $7{\cdot}44 \times 10^{-1}$)	-11·19 (-17·58 to -4·30)	0·0019
Fosphenytoin	$1{\cdot}02 \times 10^{-9}$ ($1{\cdot}41 \times 10^{-10}$ to $7{\cdot}43 \times 10^{-9}$)	$4{\cdot}08 \times 10^{-10}$ ($6{\cdot}72 \times 10^{-11}$ to $2{\cdot}48 \times 10^{-9}$)	-8·66 (-15·59 to -1·16)	0·024
Trimethadione	$1{\cdot}83 \times 10^{-11}$ ($9{\cdot}23 \times 10^{-12}$ to $3{\cdot}62 \times 10^{-11}$)	$1{\cdot}66 \times 10^{-11}$ ($8{\cdot}77 \times 10^{-12}$ to $3{\cdot}14 \times 10^{-11}$)	-0·97 (-1·28 to -0·67)	<0·0001
Ethosuximide	$4{\cdot}87 \times 10^{-6}$ ($3{\cdot}33 \times 10^{-7}$ to $7{\cdot}12 \times 10^{-5}$)	$3{\cdot}78 \times 10^{-6}$ ($2{\cdot}42 \times 10^{-7}$ to $5{\cdot}88 \times 10^{-5}$)	-2·48 (-12·88 to +9·16)	0·66
Mesuximide	$9{\cdot}24 \times 10^{-11}$ ($2{\cdot}26 \times 10^{-11}$ to $3{\cdot}78 \times 10^{-10}$)	$6{\cdot}41 \times 10^{-11}$ ($1{\cdot}71 \times 10^{-11}$ to $2{\cdot}40 \times 10^{-10}$)	-3·42 (-11·00 to +4·82)	0·40
Clonazepam	$1{\cdot}86 \times 10^{0}$ ($8{\cdot}54 \times 10^{-1}$ to $4{\cdot}06 \times 10^{0}$)	$1{\cdot}08 \times 10^{0}$ ($3{\cdot}11 \times 10^{-1}$ to $3{\cdot}78 \times 10^{0}$)	-5·24 (-12·20 to +2·27)	0·17
Carbamazepine	$7{\cdot}19 \times 10^{0}$ ($5{\cdot}63 \times 10^{0}$ to $9{\cdot}18 \times 10^{0}$)	$6{\cdot}41 \times 10^{0}$ ($5{\cdot}18 \times 10^{0}$ to $7{\cdot}93 \times 10^{0}$)	-1·09 (-1·95 to -0·23)	0·013
Oxcarbazepine	$5{\cdot}69 \times 10^{-2}$ ($7{\cdot}97 \times 10^{-3}$ to $4{\cdot}06 \times 10^{-1}$)	$2{\cdot}40 \times 10^{-1}$ ($5{\cdot}12 \times 10^{-2}$ to $1{\cdot}13 \times 10^{-0}$)	+15·44 (+2·38 to +30·16)	0·019
Rufinamide	$1{\cdot}87 \times 10^{-8}$ ($1{\cdot}55 \times 10^{-9}$ to $2{\cdot}27 \times 10^{-7}$)	$1{\cdot}10 \times 10^{-6}$ ($7{\cdot}67 \times 10^{-8}$ to $1{\cdot}58 \times 10^{-5}$)	+50·35 (+25·32 to +80·39)	<0·0001
Eslicarbazepine	$8{\cdot}44 \times 10^{-10}$ ($1{\cdot}02 \times 10^{-10}$ to $7{\cdot}01 \times 10^{-9}$)	$1{\cdot}10 \times 10^{-7}$ ($7{\cdot}16 \times 10^{-9}$ to $1{\cdot}69 \times 10^{-6}$)	+62·64 (+30·83 to +102·18)	<0·0001
Valproic acid (valproate)	$8{\cdot}95 \times 10^{0}$ ($6{\cdot}83 \times 10^{0}$ to $1{\cdot}17 \times 10^{1}$)	$1{\cdot}09 \times 10^{1}$ ($8{\cdot}68 \times 10^{0}$ to $1{\cdot}38 \times 10^{1}$)	+2·02 (+1·12 to +2·92)	<0·0001
Valpromide	$1{\cdot}30 \times 10^{-10}$ ($2{\cdot}61 \times 10^{-11}$ to $6{\cdot}48 \times 10^{-10}$)	$7{\cdot}77 \times 10^{-11}$ ($1{\cdot}87 \times 10^{-11}$ to $3{\cdot}23 \times 10^{-10}$)	-5·53 (-10·77 to +0·01)	0·050

Aminobutyric acid	1·24 × 10^{-9} (1·56 × 10^{-10} to 9·80 × 10^{-9})	1·57 × 10^{-9} (1·79 × 10^{-10} to 1·37 × 10^{-8})	+1·72 (-9·11 to +13·84)	0·77
Vigabatrin	7·37 × 10^{-5} (5·82 × 10^{-6} to 9·33 × 10^{-4})	9·31 × 10^{-4} (1·09 × 10^{-4} to 7·98 × 10^{-3})	+28·90 (+8·94 to +52·53)	0·0032
Tiagabine	1·41 × 10^{-8} (1·26 × 10^{-9} to 1·59 × 10^{-7})	5·30 × 10^{-10} (7·60 × 10^{-11} to 3·69 × 10^{-9})	-27·98 (-38·02 to -16·31)	<0·0001
Sultiame	2·11 × 10^{-9} (2·27 × 10^{-10} to 1·95 × 10^{-8})	1·83 × 10^{-8} (1·52 × 10^{-9} to 2·21 × 10^{-7})	+24·03 (+8·07 to +42·34)	0·0022
Lamotrigine	2·10 × 10^{0} (1·35 × 10^{0} to 3·26 × 10^{0})	4·32 × 10^{0} (2·96 × 10^{0} to 6·32 × 10^{0})	+7·48 (+6·34 to +8·63)	<0·0001
Felbamate	3·16 × 10^{-9} (3·43 × 10^{-10} to 2·91 × 10^{-8})	3·51 × 10^{-9} (3·79 × 10^{-10} to 3·26 × 10^{-8})	+1·04 (-11·14 to +14·90)	0·87
Topiramate	6·80 × 10^{-1} (3·17 × 10^{-1} to 1·46 × 10^{0})	9·16 × 10^{-1} (4·31 × 10^{-1} to 1·94 × 10^{0})	+1·31 (-3·91 to +6·81)	0·63
Levetiracetam	1·21 × 10^{0} (4·97 × 10^{-1} to 2·92 × 10^{0})	8·59 × 10^{0} (6·30 × 10^{0} to 1·17 × 10^{1})	+21·72 (+13·86 to +30·11)	<0·0001
Zonisamide	5·78 × 10^{-7} (3·53 × 10^{-8} to 9·48 × 10^{-6})	2·72 × 10^{-5} (1·66 × 10^{-6} to 4·47 × 10^{-4})	+47·09 (+22·15 to +77·13)	0·0001
Stiripentol	1·48 × 10^{-9} (1·87 × 10^{-10} to 1·17 × 10^{-8})	7·97 × 10^{-8} (6·36 × 10^{-9} to 9·98 × 10^{-7})	+48·97 (+22·36 to +81·38)	0·0001
Lacosamide	2·63 × 10^{-6} (1·59 × 10^{-7} to 4·33 × 10^{-5})	6·41 × 10^{-3} (6·01 × 10^{-4} to 6·84 × 10^{-2})	+116·54 (+69·22 to +177·09)	<0·0001
Retigabine	9·32 × 10^{-9} (8·90 × 10^{-10} to 9·76 × 10^{-8})	1·59 × 10^{-11} (8·84 × 10^{-12} to 2·85 × 10^{-11})	-48·67 (-58·46 to -36·58)	<0·0001
Perampanel	3·86 × 10^{-11} (1·29 × 10^{-11} to 1·15 × 10^{-10})	1·68 × 10^{-4} (1·45 × 10^{-5} to 1·95 × 10^{-3})	+357·62 (+244·22 to +508·39)	<0·0001
Brivaracetam	NA	1·65 × 10^{-5} (9·22 × 10^{-7} to 2·93 × 10^{-4})	+325·32 (+225·07 to +456·50)	<0·0001
Cannabidiol	NA	1·46 × 10^{-8} (1·35 × 10^{-9} to 1·57 × 10^{-7})	+100·02 (+66·85 to +139·79)	<0·0001
Cenobamate	NA	2·47 × 10^{-9} (2·69 × 10^{-10} to 2·26 × 10^{-8})	+59·29 (+37·38 to +84·69)	<0·0001
Fenfluramine	NA	1·14 × 10^{-10} (2·59 × 10^{-11} to 5·00 × 10^{-10})	+21·51 (+9·79 to +34·47)	0·0002

[a]Worldwide estimates with 95% CI were calculated by pooling the estimates using meta-analysis (random-effects model).

[b]The multinational trend changes were estimated using linear mixed models, controlling for within-country correlations and assuming the correlations between years were autocorrelated.

[c]Since some DDD/TID are showing very small values, they are presented as three significant figures.

*Brivaracetam, Cannabidiol, Cenobamate and Fenfluramine yet to have recorded sales in the database in 2012.

CI = Confidence interval; DDD/TID = Defined daily dose per 10,000 inhabitants per day; NA = Not applicable.

Supplementary Table 4. Top ten multinational antiseizure medication consumption rate from 2012 to 2022.

Rank	ASM and DDD/TID in 2012 (95%CI)[a]	ASM and DDD/TID in 2013 (95%CI)[a]	ASM and DDD/TID in 2014 (95%CI)[a]	ASM and DDD/TID in 2015 (95%CI)[a]	ASM and DDD/TID in 2016 (95%CI)[a]	ASM and DDD/TID in 2017 (95%CI)[a]	ASM and DDD/TID in 2018 (95%CI)[a]	ASM and DDD/TID in 2019 (95%CI)[a]	ASM and DDD/TID in 2020 (95%CI)[a]	ASM and DDD/TID in 2021 (95%CI)[a]	ASM and DDD/TID in 2022 (95%CI)[a]
1	Valproic acid (8·95, 6·83 to 11·74)	Valproic acid (9·49, 7·29 to 12·36)	Valproic acid (9·83, 7·56 to 12·79)	Valproic acid (10·24, 7·94 to 13·20)	Valproic acid (10·09, 7·82 to 13·01)	Valproic acid (10·44, 8·15 to 13·39)	Valproic acid (10·64, 8·32 to 13·61)	Valproic acid (10·71, 8·36 to 13·73)	Valproic acid (11·16, 8·81 to 14·14)	Valproic acid (10·83, 8·58 to 13·68)	Valproic acid (10·93, 8·68 to 13·77)
2	Carbamazepine (7·19, 5·63 to 9·18)	Carbamazepine (7·14, 5·61 to 9·10)	Carbamazepine (7·20, 5·68 to 9·14)	Carbamazepine (7·15, 5·68 to 9·01)	Carbamazepine (6·91, 5·48 to 8·72)	Carbamazepine (6·90, 5·50 to 8·66)	Carbamazepine (6·82, 5·44 to 8·55)	Carbamazepine (6·77, 5·43 to 8·45)	Levetiracetam (7·23, 5·20 to 10·05)	Levetiracetam (7·74, 5·60 to 10·69)	Levetiracetam (8·59, 6·30 to 11·72)
3	Lamotrigine (2·10, 1·35 to 3·26)	Lamotrigine (2·34, 1·53 to 3·59)	Lamotrigine (2·54, 1·67 to 3·87)	Lamotrigine (2·74, 1·82 to 4·11)	Levetiracetam (4·11, 2·80 to 6·03)	Levetiracetam (4·78, 3·31 to 6·89)	Levetiracetam (5·62, 3·97 to 7·95)	Levetiracetam (5·54, 3·42 to 8·96)	Carbamazepine (7·07, 5·73 to 8·71)	Carbamazepine (6·91, 5·65 to 8·44)	Carbamazepine (6·41, 5·18 to 7·93)
4	Clonazepam (1·86, 0·85 to 4·06)	Clonazepam (1·93, 0·89 to 4·19)	Levetiracetam (2·04, 0·85 to 4·88)	Levetiracetam (2·50, 1·05 to 5·97)	Lamotrigine (2·91, 1·94 to 4·36)	Lamotrigine (3·08, 2·06 to 4·60)	Lamotrigine (3·29, 2·20 to 4·92)	Lamotrigine (3·51, 2·35 to 5·23)	Lamotrigine (3·87, 2·64 to 5·68)	Lamotrigine (4·13, 2·83 to 6·04)	Lamotrigine (4·32, 2·96 to 6·32)
5	Levetiracetam (1·21, 0·50 to 2·92)	Levetiracetam (1·62, 0·68 to 3·90)	Clonazepam (1·91, 0·87 to 4·22)	Clonazepam (1·73, 0·76 to 3·94)	Clonazepam (1·86, 0·83 to 4·18)	Clonazepam (1·98, 0·90 to 4·38)	Clonazepam (2·02, 0·91 to 4·45)	Clonazepam (1·97, 0·90 to 4·35)	Clonazepam (1·96, 0·87 to 4·40)	Clonazepam (1·55, 0·54 to 4·50)	Clonazepam (1·08, 0·31 to 3·78)
6	Topiramate (0·68, 0·32 to 1·46)	Topiramate (0·94, 0·64 to 1·39)	Topiramate (1·00, 0·68 to 1·46)	Topiramate (1·05, 0·72 to 1·53)	Topiramate (0·97, 0·64 to 1·49)	Topiramate (0·79, 0·37 to 1·69)	Topiramate (0·61, 0·22 to 1·71)	Topiramate (0·64, 0·23 to 1·76)	Topiramate (1·03, 0·66 to 1·62)	Topiramate (0·90, 0·42 to 1·92)	Topiramate (0·92, 0·43 to 1·94)
7	Phenobarbital (0·49, 0·11 to 2·28)	Phenobarbital (0·63, 0·16 to 2·54)	Phenobarbital (0·44, 0·10 to 2·05)	Phenytoin (0·43, 0·11 to 1·77)	Phenytoin (0·41, 0·10 to 1·65)	Phenobarbital (0·52, 0·13 to 2·04)	Phenobarbital (0·50, 0·13 to 1·96)	Phenobarbital (0·45, 0·11 to 1·78)	Phenobarbital (0·33, 0·07 to 1·51)	Phenobarbital (0·49, 0·15 to 1·66)	Phenobarbital (0·36, 0·09 to 1·45)
8	Phenytoin (0·45, 0·09 to 2·12)	Phenytoin (0·43, 0·09 to 2·02)	Phenytoin (0·28, 0·05 to 1·47)	Phenobarbital (0·42, 0·09 to 1·91)	Phenobarbital (0·39, 0·09 to 1·81)	Phenytoin (0·30, 0·06 to 1·41)	Phenytoin (0·28, 0·06 to 1·31)	Oxcarbazepine (0·23, 0·05 to 1·06)	Oxcarbazepine (0·22, 0·05 to 1·06)	Oxcarbazepine (0·24, 0·05 to 1·13)	Oxcarbazepine (0·24, 0·05 to 1·13)
9	Oxcarbazepine (0·06, 0·01 to 0·41)	Oxcarbazepine (0·14, 0·03 to 0·75)	Oxcarbazepine (0·15, 0·03 to 0·81)	Oxcarbazepine (0·16, 0·03 to 0·84)	Oxcarbazepine (0·13, 0·02 to 0·75)	Oxcarbazepine (0·21, 0·04 to 0·99)	Oxcarbazepine (0·22, 0·05 to 1·05)	Phenytoin (0·20, 0·04 to 1·07)	Phenytoin (0·20, 0·04 to 1·05)	Phenytoin (0·19, 0·04 to 0·96)	Phenytoin (0·13, 0·02 to 0·74)
10	Vigabatrin (0·00, 0·00 to 0·00)	Vigabatrin (0·00, 0·00 to 0·00)	Lacosamide (0·00, 0·00 to 0·00)	Lacosamide (0·00, 0·00 to 0·00)	Lacosamide (0·00, 0·00 to 0·01)	Lacosamide (0·00, 0·00 to 0·01)	Lacosamide (0·00, 0·00 to 0·01)	Lacosamide (0·00, 0·00 to 0·02)	Lacosamide (0·00, 0·00 to 0·03)	Lacosamide (0·00, 0·00 to 0·04)	Lacosamide (0·01, 0·00 to 0·01)

[a]Worldwide estimates with 95% CI were calculated by pooling the estimates using meta-analysis (random-effects model).

ASM = Antiseizure medication; CI = Confidence interval; DDD/TID = Defined daily dose per 10,000 inhabitants per day

Supplementary Table 5. Multinational, regional, and national levels of valproate consumption rate in 2012 and 2022, and their average annual percentage change.

Country/ region	DDD/TID in 2012 (95%CI)[a]	DDD/TID in 2022 (95%CI)[a]	Average annual percentage change (%, 95%CI)[b]	P value
**Multinational**	8·95 (6·83 to 11·74)	10·93 (8·68 to 13·77)	+2·02 (+1·12 to +2·92)	<0·0001
**Northern America**	21·19 (14·56 to 30·83)	18·08 (5·96 to 54·84)	−1·57 (−2·58 to −0·56)	0·0044
Canada	21·82 (21·82 to 21·83)	19·73 (19·72 to 19·74)	-0·82 (-1·10 to -0·55)	0·0001
United States	20·57 (20·57 to 20·58)	16·57 (16·57 to 16·57)	-1·84 (-2·33 to -1·35)	<0·0001
**Central and South America and the Caribbean**	4·00 (2·09 to 7·66)	4·26 (2·21 to 8·22)	+0·63 (−1·90 to +3·23)	0·63
Argentina	11·74 (11·74 to 11·75)	10·54 (10·53 to 10·54)	-1·82 (-3·24 to -0·37)	0·019
Brazil	4·59 (4·59 to 4·59)	6·49 (6·49 to 6·50)	+3·62 (+2·74 to +4·50)	<0·0001
Chile	1·96 (1·95 to 1·96)	2·76 (2·75 to 2·76)	+3·54 (+1·92 to +5·18)	0·0007
Colombia	1·00 (1·00 to 1·00)	1·13 (1·13 to 1·13)	+1·59 (+0·40 to +2·80)	0·015
Dominican Republic	3·08 (3·07 to 3·08)	4·74 (4·74 to 4·75)	+4·58 (+2·99 to +6·20)	0·0001
Ecuador	3·03 (3·02 to 3·03)	4·32 (4·32 to 4·33)	+2·81 (-0·14 to +5·85)	0·060
Mexico	2·32 (2·31 to 2·32)	2·60 (2·60 to 2·60)	+0·79 (+0·13 to +1·46)	0·025
Peru	1·21 (1·21 to 1·21)	1·47 (1·46 to 1·47)	+1·96 (-0·19 to +4·15)	0·070
Puerto Rico	20·50 (20·48 to 20·52)	25·09 (25·06 to 25·12)	+2·41 (+1·30 to +3·53)	0·0008
Uruguay	9·72 (9·71 to 9·74)	12·39 (12·37 to 12·41)	+2·91 (+2·07 to +3·75)	<0·0001
Venezuela	7·64 (7·64 to 7·65)	1·63 (1·63 to 1·63)	-18·42 (-24·00 to -12·42)	0·0001
**Western Europe**	26·96 (17·33 to 41·95)	22·40 (14·12 to 35·54)	−1·84 (−2·97 to −0·69)	0·0022
Austria	16·68 (16·66 to 16·69)	18·30 (18·28 to 18·31)	+0·94 (+0·20 to +1·68)	0·019
Belgium	71·64 (71·61 to 71·66)	64·80 (64·78 to 64·83)	-0·42 (-2·74 to +1·96)	0·70
France	31·55 (31·54 to 31·55)	24·72 (24·71 to 24·73)	-2·50 (-3·11 to -1·89)	<0·0001
Germany	22·60 (22·59 to 22·60)	18·01 (18·00 to 18·01)	-2·32 (-2·52 to -2·12)	<0·0001
Luxembourg	22·05 (21·99 to 22·12)	14·36 (14·31 to 14·41)	-4·23 (-4·47 to -3·98)	<0·0001
Netherlands	27·52 (27·50 to 27·53)	21·70 (21·69 to 21·71)	-2·67 (-3·11 to -2·23)	<0·0001
Switzerland	20·04 (20·02 to 20·05)	17·23 (17·21 to 17·24)	-1·59 (-1·88 to -1·31)	<0·0001
**Northern Europe**	20·23 (13·90 to 29·44)	20·94 (16·70 to 26·26)	+0·34 (−1·27 to +1·98)	0·68

Estonia	13·11 (13·08 to 13·14)	16·33 (16·29 to 16·36)	+1·45 (+0·58 to +2·34)	0·0043
Finland	36·66 (36·64 to 36·69)	33·13 (33·11 to 33·16)	-1·03 (-1·39 to -0·67)	0·0001
Ireland	36·59 (36·56 to 36·62)	24·66 (24·64 to 24·68)	-5·35 (-7·41 to -3·24)	0·0003
Latvia	11·10 (11·07 to 11·12)	20·55 (20·51 to 20·58)	+4·87 (+2·03 to +7·79)	0·0036
Lithuania	18·12 (18·09 to 18·14)	23·17 (23·14 to 23·20)	+3·09 (+1·78 to +4·43)	0·0005
Norway	17·30 (17·29 to 17·32)	17·51 (17·49 to 17·53)	-0·05 (-0·27 to +0·18)	0·65
Sweden	16·88 (16·87 to 16·89)	14·08 (14·07 to 14·10)	-1·69 (-2·16 to -1·21)	<0·0001
United Kingdom	27·18 (27·18 to 27·19)	23·59 (23·58 to 23·59)	-1·52 (-2·28 to -0·76)	0·0014
**Southern Europe**	17·62 (11·86 to 26·16)	22·12 (16·82 to 29·09)	+2·30 (+1·41 to +3·21)	<0·0001
Bosnia And Herzegovina	6·29 (6·28 to 6·30)	13·12 (13·10 to 13·14)	+6·44 (+4·98 to +7·92)	<0·0001
Croatia	16·02 (16·00 to 16·04)	19·85 (19·83 to 19·87)	+1·86 (+1·55 to +2·17)	<0·0001
Greece	18·40 (18·39 to 18·42)	26·97 (26·95 to 26·98)	+3·61 (+2·80 to +4·43)	<0·0001
Italy	22·11 (22·10 to 22·11)	26·35 (26·35 to 26·36)	+1·61 (+1·14 to +2·10)	<0·0001
Portugal	32·60 (32·58 to 32·62)	32·34 (32·32 to 32·36)	−0·14 (−0·57 to +0·28)	0·46
Serbia	20·82 (20·80 to 20·83)	31·29 (31·26 to 31·31)	+4·67 (+3·97 to +5·38)	<0·0001
Slovenia	16·04 (16·02 to 16·07)	15·76 (15·73 to 15·79)	−0·40 (−1·02 to +0·23)	0·19
Spain	20·79 (20·78 to 20·80)	19·43 (19·42 to 19·44)	−0·59 (−1·23 to +0·06)	0·071
**Eastern Europe**	14·61 (8·38 to 25·49)	20·48 (13·96 to 30·04)	+3·43 (+2·17 to +4·72)	<0·0001
Belarus	5·70 (5·70 to 5·71)	12·19 (12·17 to 12·20)	+8·11 (+7·20 to +9·02)	<0·0001
Bulgaria	22·41 (22·39 to 22·43)	31·50 (31·48 to 31·52)	+4·21 (+3·13 to +5·31)	<0·0001
Czech Republic	19·03 (19·02 to 19·04)	20·62 (20·61 to 20·63)	+0·46 (-0·70 to +1·64)	0·39
Hungary	11·96 (11·95 to 11·97)	14·64 (14·63 to 14·65)	+1·82 (+1·21 to +2·43)	<0·0001
Poland	28·70 (28·69 to 28·71)	34·67 (34·66 to 34·68)	+1·48 (+0·48 to +2·49)	0·0084
Romania	21·82 (21·81 to 21·83)	27·66 (27·64 to 27·67)	+1·77 (+0·75 to +2·81)	0·0035
Russia	5·07 (5·07 to 5·08)	10·38 (10·38 to 10·39)	+6·10 (+3·80 to +8·46)	0·0002
Slovakia	22·50 (22·48 to 22·52)	26·84 (26·81 to 26·86)	+1·59 (+1·33 to +1·85)	<0·0001
**Oceania**	37·07 (25·34 to 54·23)	30·11 (12·55 to 72·23)	−2·06 (−2·78 to −1·33)	<0·0001
Australia	38·20 (38·18 to 38·21)	32·26 (32·24 to 32·27)	-1·70 (-1·93 to -1·48)	<0·0001
New Zealand	35·98 (35·95 to 36·01)	28·11 (28·08 to 28·13)	-2·56 (-2·97 to -2·14)	<0·0001
**Eastern Asia**	10·25 (2·65 to 39·62)	12·56 (4·76 to 33·12)	+2·05 (+0·71 to +3·42)	0·0034

China	1·54 (1·54 to 1·54)	3·19 (3·19 to 3·19)	+8·20 (+7·24 to +9·18)	<0·0001
Hong Kong	16·16 (16·15 to 16·18)	18·63 (18·61 to 18·64)	+1·70 (+0·54 to +2·87)	0·0089
Japan	24·95 (24·95 to 24·96)	21·56 (21·56 to 21·56)	-1·57 (-2·16 to -0·98)	0·0002
South Korea	13·43 (13·42 to 13·43)	14·28 (14·27 to 14·28)	+0·55 (+0·20 to +0·89)	0·0056
Taiwan	13·57 (13·56 to 13·57)	17·11 (17·10 to 17·12)	+2·07 (+1·46 to +2·68)	<0·0001
**Central Asia**	6·29 (6·28 to 6·29)	7·81 (7·80 to 7·81)	+2·13 (-1·11 to +5·49)	0·17
Kazakhstan	6·29 (6·28 to 6·29)	7·81 (7·80 to 7·81)	+2·07 (+0·18 to +4·00)	0·035
**Western Asia**	3·22 (1·03 to 10·11)	4·65 (1·60 to 13·53)	+3·73 (−2·45 to +10·30)	0·24
Jordan	2·26 (2·25 to 2·26)	3·00 (3·00 to 3·01)	+1·31 (-0·48 to +3·13)	0·13
Kuwait	0·68 (0·67 to 0·68)	1·13 (1·12 to 1·13)	+5·60 (+0·81 to +10·61)	0·026
Lebanon	8·60 (8·58 to 8·61)	5·02 (5·02 to 5·03)	-0·29 (-8·27 to +8·38)	0·94
Saudi Arabia	3·04 (3·04 to 3·05)	13·34 (13·33 to 13·34)	+12·35 (+4·12 to +21·24)	0·0071
Türkiye	14·10 (14·10 to 14·11)	16·33 (16·32 to 16·33)	+1·72 (+0·62 to +2·82)	0·0061
United Arab Emirates	1·99 (1·98 to 1·99)	2·73 (2·72 to 2·73)	+2·06 (-1·96 to +6·23)	0·28
**South-eastern Asia**	1·96 (0·46 to 8·30)	3·61 (1·11 to 11·79)	+6·33 (+3·58 to +9·15)	<0·0001
Indonesia	0·30 (0·30 to 0·30)	0·63 (0·63 to 0·63)	+9·66 (+3·49 to +16·19)	0·0057
Malaysia	6·51 (6·51 to 6·52)	7·74 (7·74 to 7·75)	+1·84 (−0·88 to +4·64)	0·16
Philippines	0·64 (0·64 to 0·64)	1·78 (1·78 to 1·78)	+9·72 (+7·91 to +11·56)	<0·0001
Singapore	8·46 (8·45 to 8·48)	11·57 (11·55 to 11·58)	+2·69 (+1·97 to +3·41)	<0·0001
Thailand	4·76 (4·76 to 4·76)	8·67 (8·66 to 8·67)	+6·59 (+5·37 to +7·83)	<0·0001
Vietnam	1·13 (1·13 to 1·13)	2·54 (2·54 to 2·54)	+8·81 (+5·84 to +11·87)	0·0001
**Southern Asia**	2·86 (1·60 to 5·10)	4·94 (3·76 to 6·51)	+5·65 (+3·01 to +8·35)	0·0001
Bangladesh	1·66 (1·66 to 1·66)	4·50 (4·50 to 4·50)	+10·52 (+8·22 to +12·87)	0·0001
India	3·56 (3·56 to 3·56)	4·52 (4·52 to 4·52)	+3·08 (+2·49 to +3·69)	<0·0001
Pakistan	3·24 (3·24 to 3·24)	6·40 (6·40 to 6·40)	+6·61 (+5·40 to +7·84)	<0·0001
Sri Lanka	3·48 (3·47 to 3·48)	4·59 (4·58 to 4·59)	+3·18 (+0·76 to +5·65)	0·015
**Northern Africa**	8·35 (2·46 to 28·36)	11·31 (4·73 to 27·01)	+3·08 (-0·15 to +6·42)	0·061
Algeria	11·48 (11·47 to 11·48)	11·54 (11·53 to 11·54)	+0·73 (-1·75 to +3·26)	0·53
Egypt	4·83 (4·83 to 4·84)	10·65 (10·65 to 10·66)	+9·78 (+7·66 to +11·94)	<0·0001

Morocco	4·13 (4·13 to 4·13)	5·91 (5·90 to 5·91)	+3·33 (+2·61 to +4·05)	<0·0001
Tunisia	21·27 (21·26 to 21·28)	22·50 (22·48 to 22·51)	-0·57 (-2·29 to +1·19)	0·48
**Southern Africa**	14·57 (14·57 to 14·58)	27·26 (27·25 to 27·27)	+6·08 (-0·78 to +13·41)	0·077
South Africa	14·57 (14·57 to 14·58)	27·26 (27·25 to 27·27)	+6·04 (+0·04 to +12·40)	0·049

[a]Worldwide and regional estimates with 95% CI were calculated by pooling the estimates using meta-analysis (random-effects model).

[b]The average annual percentage change is calculated using a linear regression model, with log-transformed consumption in DDD/TID as the dependent variable and year as the independent variable. The average annual change was expressed as average annual percentage change, calculated by [exp(the coefficient of the year variable) - 1] x 100%. The multinational and regional trend changes were estimated using linear mixed models, controlling for within-country correlations and assuming the correlations between years were autocorrelated.

CI = Confidence interval; DDD/TID = Defined daily dose per 10,000 inhabitants per day.

Supplementary Table 6. Annual pooled valproate consumption rate and average annual percentage change from 2012 to 2022 by country income level.

Income level	High (n=41)	Upper-Middle (n=20)	Lower-Middle (n=12)
Average annual percentage change[a] (%, 95% CI)	+0·86 (−0·07 to +1·79)	+3·10 (+0·95 to +5·29)	+4·24 (+1·73 to +6·81)
**Year**	**Pooled consumption rate (DDD/TID)**		
2012	16·22 (12·31 to 21·37)	4·59 (2·76 to 7·63)	3·59 (1·93 to 6·68)
2013	16·89 (12·88 to 22·15)	4·91 (2·95 to 8·16)	3·98 (2·25 to 7·06)
2014	17·70 (13·75 to 22·78)	5·03 (2·98 to 8·51)	4·02 (2·25 to 7·19)
2015	17·59 (13·55 to 22·83)	5·58 (3·36 to 9·29)	4·42 (2·58 to 7·60)
2016	17·32 (13·22 to 22·70)	5·20 (3·19 to 8·49)	4·79 (2·79 to 8·24)
2017	17·81 (13·84 to 22·91)	5·57 (3·38 to 9·17)	4·81 (2·81 to 8·23)
2018	17·68 (13·68 to 22·86)	5·85 (3·51 to 9·74)	5·09 (3·08 to 8·42)
2019	17·67 (13·68 to 22·82)	5·77 (3·39 to 9·82)	5·43 (3·24 to 9·10)
2020	17·97 (14·21 to 22·72)	6·16 (3·64 to 10·41)	5·91 (3·56 to 9·81)
2021	17·69 (14·15 to 22·11)	5·98 (3·55 to 10·09)	5·46 (3·45 to 8·63)
2022	17·66 (14·13 to 22·07)	6·22 (3·70 to 10·48)	5·44 (3·49 to 8·47)

[a]The average annual change is calculated using a linear regression model, with log-transformed consumption rate in DDD/TID as the dependent variable and year as the independent variable. The average annual change was expressed as average annual percentage change, calculated by [exp(the coefficient of the year variable) - 1] x 100%.

CI = Confidence interval; DDD/TID = Defined daily dose per 10,000 inhabitants per day.

Supplementary Table 7. Multinational, regional, and national levels of antiseizure medication consumption rate as DDD/HED in 2012 and 2021 and average annual percentage change.

Country/ region*	DDD/HED in 2012 (95%CI)[a]	DDD/HED in 2021 (95%CI)[a]	Average annual percentage change (%, 95%CI)[b]	P value
**Multinational**	57·63 (44·62 to 74·44)	74·40 (58·75 to 94·22)	+2·88 (+2·09 to +3·67)	<0·0001
**Northern America**	205·20 (125·71 to 334·95)	232·32 (181·77 to 296·92)	+1·35 (+0·45 to +2·27)	0·0056
Canada	213·27 (213·23 to 213·30)	227·88 (227·84 to 227·91)	+0·40 (−0·07 to +0·86)	0·085
United States	197·44 (197·43 to 197·45)	236·85 (236·84 to 236·86)	+1·83 (+1·13 to +2·54)	0·0003
**Central and South America and the Caribbean**	34·68 (17·09 to 70·40)	37·32 (17·98 to 77·44)	+0·82 (-1·98 to +3·70)	0·57
Argentina	151·29 (151·26 to 151·31)	163·43 (163·41 to 163·46)	+0·29 (−0·53 to +1·11)	0·45
Brazil	65·35 (65·35 to 65·36)	85·97 (85·97 to 85·98)	+3·41 (+2·67 to +4·16)	<0·0001
Chile	30·60 (30·59 to 30·61)	37·44 (37·42 to 37·45)	+1·64 (+0·76 to +2·53)	0·0026
Colombia	8·72 (8·71 to 8·72)	8·83 (8·83 to 8·83)	+1·23 (−0·44 to +2·92)	0·13
Dominican Republic	17·84 (17·83 to 17·86)	25·82 (25·80 to 25·84)	+5·12 (+3·02 to +7·26)	0·0004
Ecuador	18·35 (18·34 to 18·36)	22·85 (22·84 to 22·86)	+2·01 (−0·54 to +4·61)	0·11
Mexico	13·24 (13·24 to 13·24)	15·26 (15·26 to 15·26)	+1·90 (+0·83 to +2·98)	0·0034
Peru	9·93 (9·93 to 9·94)	15·74 (15·73 to 15·74)	+4·77 (+1·48 to +8·18)	0·0099
Puerto Rico	134·47 (134·41 to 134·53)	174·57 (174·49 to 174·64)	+2·30 (+1·35 to +3·25)	0·0005
Uruguay	105·46 (105·39 to 105·52)	127·10 (127·03 to 127·17)	+1·65 (+0·92 to +2·38)	0·0008
Venezuela	54·24 (54·22 to 54·25)	13·39 (13·38 to 13·40)	−18·93 (−27·54 to −9·30)	0·0026
**Western Europe**	140·67 (108·64 to 182·15)	157·46 (124·44 to 199·24)	+1·26 (+0·67 to +1·85)	0·0001
Austria	117·17 (117·13 to 117·22)	139·89 (139·84 to 139·93)	+2·05 (+1·58 to +2·52)	<0·0001
Belgium	244·51 (244·45 to 244·57)	271·78 (271·72 to 271·85)	+1·83 (+0·99 to +2·66)	0·0009

France	148·39 (148·37 to 148·41)	157·89 (157·87 to 157·91)	+0·57 (+0·25 to +0·90)	0·0034
Germany	98·98 (98·97 to 98·99)	123·10 (123·09 to 123·12)	+2·59 (+1·80 to +3·39)	0·0001
Luxembourg	134·37 (134·17 to 134·56)	141·38 (141·20 to 141·57)	+0·60 (+0·34 to +0·87)	0·0007
Netherlands	141·08 (141·04 to 141·12)	147·61 (147·57 to 147·65)	+0·40 (+0·17 to +0·62)	0·0035
Switzerland	136·65 (136·60 to 136·71)	155·62 (155·57 to 155·68)	+1·56 (+1·08 to +2·05)	0·0001
**Northern Europe**	121·37 (78·88 to 186·74)	153·49 (107·00 to 220·19)	+2·64 (+1·54 to +3·76)	<0·0001
Estonia	63·06 (62·99 to 63·13)	77·14 (77·06 to 77·22)	+2·19 (+1·78 to +2·60)	<0·0001
Finland	230·19 (230·10 to 230·28)	264·76 (264·66 to 264·86)	+1·59 (+1·35 to +1·83)	<0·0001
Ireland	196·65 (196·56 to 196·73)	226·80 (226·71 to 226·89)	+1·41 (+0·44 to +2·39)	0·010
Latvia	56·59 (56·53 to 56·65)	101·60 (101·52 to 101·69)	+5·41 (+2·92 to +7·95)	0·0009
Lithuania	90·58 (90·52 to 90·64)	110·63 (110·56 to 110·70)	+1·63 (−0·45 to +3·76)	0·11
Norway	141·23 (141·17 to 141·29)	160·13 (160·06 to 160·19)	+1·57 (+1·32 to +1·82)	<0·0001
Sweden	156·26 (156·20 to 156·31)	195·53 (195·47 to 195·59)	+2·94 (+2·43 to +3·46)	<0·0001
United Kingdom	145·82 (145·80 to 145·84)	189·00 (188·97 to 189·02)	+3·24 (+2·57 to +3·91)	<0·0001
**Southern Europe**	124·05 (75·63 to 203·47)	171·05 (111·98 to 261·30)	+3·63 (+2·77 to +4·51)	<0·0001
Bosnia And Herzegovina	51·22 (51·18 to 51·26)	85·55 (85·50 to 85·61)	+5·79 (+4·48 to +7·10)	<0·0001
Croatia	76·85 (76·81 to 76·89)	101·46 (101·41 to 101·51)	+3·22 (+2·84 to +3·60)	<0·0001
Greece	145·54 (145·50 to 145·59)	233·53 (233·47 to 233·59)	+5·58 (+4·94 to +6·22)	<0·0001
Italy	230·96 (230·93 to 230·99)	252·26 (252·23 to 252·29)	+1·01 (+0·48 to +1·54)	0·0022
Portugal	261·82 (261·75 to 261·90)	330·42 (330·34 to 330·51)	+2·91 (+2·41 to +3·41)	<0·0001

Serbia	86·16 (86·13 to 86·19)	134·11 (134·06 to 134·15)	+5·62 (+4·35 to +6·91)	<0·0001
Slovenia	92·01 (91·93 to 92·09)	122·15 (122·06 to 122·24)	+3·18 (+2·72 to +3·65)	<0·0001
Spain	204·23 (204·20 to 204·26)	264·81 (264·78 to 264·84)	+3·13 (+2·73 to +3·54)	<0·0001
**Eastern Europe**	77·17 (55·57 to 107·17)	99·17 (77·18 to 127·43)	+2·83 (+1·86 to +3·81)	<0·0001
Belarus	46·81 (46·78 to 46·84)	72·58 (72·55 to 72·62)	+5·62 (+4·84 to +6·41)	<0·0001
Bulgaria	88·38 (88·34 to 88·42)	111·23 (111·18 to 111·28)	+2·41 (+1·79 to +3·04)	<0·0001
Czech Republic	97·94 (97·91 to 97·97)	127·54 (127·50 to 127·58)	+2·71 (+1·83 to +3·60)	0·0001
Hungary	124·60 (124·56 to 124·63)	132·37 (132·33 to 132·41)	+0·51 (-0·13 to +1·15)	0·11
Poland	114·38 (114·36 to 114·41)	142·90 (142·88 to 142·92)	+2·40 (+1·52 to +3·30)	0·0002
Romania	77·75 (77·73 to 77·77)	91·15 (91·13 to 91·17)	+1·51 (+0·68 to +2·33)	0·0028
Russia	43·01 (43·00 to 43·02)	63·36 (63·35 to 63·36)	+4·59 (+3·09 to +6·12)	0·0001
Slovakia	65·15 (65·12 to 65·19)	83·18 (83·15 to 83·22)	+2·26 (+1·22 to +3·30)	0·0010
**Oceania**	177·37 (122·58 to 256·64)	192·72 (124·13 to 299·19)	+0·93 (+0·62 to +1·23)	<0·0001
Australia	182·60 (182·56 to 182·64)	199·50 (199·47 to 199·54)	+0·94 (+0·66 to +1·21)	<0·0001
New Zealand	172·28 (172·20 to 172·37)	186·16 (186·07 to 186·24)	+0·83 (+0·63 to +1·02)	<0·0001
**Eastern Asia**	59·87 (10·15 to 353·02)	81·25 (16·55 to 398·95)	+3·45 (+2·27 to +4·64)	<0·0001
China	12·08 (12·08 to 12·08)	19·42 (19·42 to 19·42)	+6·05 (+4·83 to +7·28)	<0·0001
Japan	160·27 (160·26 to 160·29)	195·34 (195·32 to 195·36)	+2·39 (+1·96 to +2·83)	<0·0001
South Korea	76·48 (76·47 to 76·50)	95·11 (95·09 to 95·13)	+2·48 (+2·10 to +2·86)	<0·0001
Taiwan	86·80 (86·77 to 86·82)	120·79 (120·77 to 120·82)	+3·68 (+3·32 to +4·03)	<0·0001
**Central Asia**	21·07 (21·06 to 21·08)	27·87 (27·85 to 27·88)	+3·42 (+2·20 to +4·66)	0·0002
Kazakhstan	21·07 (21·06 to 21·08)	27·87 (27·85 to 27·88)	+3·43 (+2·26 to +4·62)	0·0001
**Western Asia**	19·64 (5·08 to 75·93)	34·67 (11·44 to 105·08)	+6·57 (+2·94 to +10·33)	0·0005

Jordan	13·59 (13·58 to 13·61)	18·22 (18·21 to 18·24)	+2·88 (−0·09 to +5·94)	0·056
Kuwait	3·26 (3·25 to 3·27)	6·68 (6·67 to 6·70)	+9·66 (+3·34 to +16·35)	0·0071
Lebanon	97·89 (97·83 to 97·96)	90·31 (90·26 to 90·37)	+1·76 (−0·88 to +4·47)	0·17
Saudi Arabia	18·38 (18·37 to 18·39)	51·13 (51·11 to 51·14)	+11·39 (+2·62 to +20·91)	0·016
Türkiye	75·88 (75·87 to 75·89)	110·53 (110·52 to 110·55)	+4·23 (+3·38 to +5·10)	<0·0001
United Arab Emirates	9·48 (9·47 to 9·49)	27·95 (27·93 to 27·97)	+10·56 (+7·33 to +13·89)	0·0001
**South-eastern Asia**	14·44 (4·80 to 43·41)	22·32 (8·30 to 60·04)	+5·11 (+3·08 to +7·17)	<0·0001
Indonesia	4·57 (4·57 to 4·57)	7·86 (7·85 to 7·86)	+7·92 (+2·29 to +13·86)	0·011
Malaysia	24·72 (24·71 to 24·73)	27·82 (27·81 to 27·83)	+1·16 (-0·44 to +2·78)	0·13
Philippines	6·24 (6·24 to 6·24)	10·65 (10·65 to 10·66)	+6·01 (+5·16 to +6·86)	<0·0001
Singapore	50·57 (50·52 to 50·61)	69·04 (68·99 to 69·09)	+2·97 (+2·18 to +3·77)	<0·0001
Thailand	38·94 (38·93 to 38·95)	63·83 (63·82 to 63·85)	+5·47 (+4·13 to +6·81)	<0·0001
Vietnam	6·54 (6·53 to 6·54)	12·06 (12·05 to 12·06)	+9·20 (+6·55 to +11·92)	<0·0001
**Southern Asia**	20·60 (11·89 to 35·71)	28·06 (12·13 to 64·94)	+3·49 (+0·86 to +6·20)	0·010
Bangladesh	19·98 (19·97 to 19·98)	39·31 (39·30 to 39·31)	+8·85 (+6·28 to +11·48)	<0·0001
India	33·90 (33·90 to 33·91)	45·98 (45·98 to 45·98)	+3·56 (+3·00 to +4·11)	<0·0001
Pakistan	16·40 (16·39 to 16·40)	23·95 (23·95 to 23·95)	+4·33 (+3·60 to +5·06)	<0·0001
Sri Lanka	16·22 (16·21 to 16·23)	14·33 (14·32 to 14·34)	−0·58 (−2·55 to +1·44)	0·53
**Northern Africa**	52·60 (16·75 to 165·25)	71·77 (29·19 to 176·49)	+3·51 (+0·93 to +6·16)	0·0087
Algeria	79·18 (79·16 to 79·20)	95·08 (95·06 to 95·10)	+2·90 (+1·79 to +4·02)	0·0003
Egypt	30·38 (30·37 to 30·38)	72·83 (72·82 to 72·84)	+11·66 (+9·09 to +14·29)	<0·0001
Morocco	27·12 (27·10 to 27·13)	32·43 (32·41 to 32·44)	+2·03 (+1·58 to +2·47)	<0·0001
Tunisia	117·41 (117·36 to 117·45)	118·17 (118·12 to 118·21)	-0·02 (-1·04 to +1·01)	0·96
**Southern Africa**	72·15 (72·13 to 72·16)	66·14 (66·13 to 66·15)	+0·25 (-4·19 to +4·89)	0·90
South Africa	72·15 (72·13 to 72·16)	66·14 (66·13 to 66·15)	+0·57 (−2·89 to +4·14)	0·72

[a]Worldwide and regional estimates with 95% CI were calculated by pooling the estimates using meta-analysis (random-effects model).

[b]The average annual percentage change is calculated using a linear regression model, with log-transformed consumption in DDD/HED as the dependent variable and year as the independent variable. The average annual

change was expressed as average annual percentage change, calculated by [exp(the coefficient of the year variable) - 1] x 100%. The multinational and regional trend changes were estimated using linear mixed models, controlling for within-country correlations and assuming the correlations between years were autocorrelated.

*Hong Kong is not included in this table since it is not included in Global Burden of Disease database for all-cause epilepsy.

#The last year of information availability from Global Burden of Disease is 2021.

CI = Confidence interval; DDD/HED = Defined daily dose per 100 epilepsy patients per day.

Supplementary Table 8. Annual pooled antiseizure medication consumption rate as DDD/HED and average annual percentage change from 2012 to 2021 by country income level.

Income level	High (n=40)*	Upper-Middle (n=20)	Lower-Middle (n=12)
Average annual percentage change[a] (%, 95% CI)	+2·87 (+2·03 to +3·71)	+3·62 (+1·76 to +5·52)	+2·45 (+0·51 to +4·44)
**Year**	**Pooled consumption (DDD/HED)**		
2012	99·47 (75·07 to 131·82)	31·57 (20·39 to 48·88)	25·48 (13·81 to 47·02)
2013	104·03 (78·47 to 137·90)	32·90 (21·31 to 50·79)	26·51 (15·01 to 46·82)
2014	109·15 (83·05 to 143·45)	33·29 (21·16 to 52·36)	26·56 (14·88 to 47·40)
2015	111·46 (84·86 to 146·40)	34·88 (22·54 to 53·99)	28·00 (16·01 to 48·98)
2016	112·32 (84·84 to 148·69)	33·52 (21·56 to 52·10)	29·80 (17·13 to 51·84)
2017	117·76 (91·06 to 152·29)	34·67 (22·14 to 54·30)	30·65 (17·66 to 53·18)
2018	118·93 (91·92 to 153·88)	36·12 (23·06 to 56·59)	32·87 (19·19 to 56·30)
2019	121·99 (94·63 to 157·26)	37·06 (23·47 to 58·50)	34·43 (20·10 to 58·97)
2020	126·51 (99·18 to 161·37)	40·15 (25·79 to 62·50)	36·77 (21·31 to 63·43)
2021	128·29 (102·00 to 161·37)	39·27 (25·25 to 61·09)	35·09 (20·46 to 60·17)

[a]The average annual change is calculated using a linear regression model, with log-transformed consumption in DDD/HED as the dependent variable and year as the independent variable. The average annual change was expressed as average annual percentage change, calculated by [exp(the coefficient of the year variable) - 1] x 100%.

[*]Hong Kong is not included in this table since it is not included in Global Burden of Disease database for all-cause epilepsy.

[#]The last year of information availability from Global Burden of Disease is 2021.

CI = Confidence interval; DDD/HED = Defined Daily Dose per 100 epilepsy patients per day.

Supplementary Table 9. Multinational levels of individual antiseizure medication consumption rate as DDD/HED between 2012 and 2021 and average annual percentage change.

Antiseizure medication	DDD/HED in 2012 (95%CI)[a,c]	DDD/HED in 2021 (95%CI)[a,c]	Average annual percentage change (%, 95%CI)[b]	P value
Methylphenobarbital	$1{\cdot}29 \times 10^{-10}$ ($2{\cdot}68 \times 10^{-11}$ to $6{\cdot}17 \times 10^{-10}$)	$8{\cdot}44 \times 10^{-11}$ ($1{\cdot}98 \times 10^{-11}$ to $3{\cdot}59 \times 10^{-10}$)	-4·10 (-7·15 to -0·96)	0·011
Phenobarbital	$6{\cdot}82 \times 10^{-1}$ ($1{\cdot}44 \times 10^{-1}$ to $3{\cdot}23 \times 10^{0}$)	$6{\cdot}85 \times 10^{-1}$ ($1{\cdot}99 \times 10^{-1}$ to $2{\cdot}36 \times 10^{0}$)	-1·48 (-7·42 to +4·85)	0·64
Primidone	$1{\cdot}21 \times 10^{-5}$ ($7{\cdot}51 \times 10^{-7}$ to $1{\cdot}97 \times 10^{-4}$)	$1{\cdot}04 \times 10^{-5}$ ($6{\cdot}45 \times 10^{-7}$ to $1{\cdot}68 \times 10^{-4}$)	-2·05 (-13·11 to +10·43)	0·74
Ethotoin	$4{\cdot}46 \times 10^{-11}$ ($1{\cdot}62 \times 10^{-11}$ to $1{\cdot}23 \times 10^{-10}$)	$2{\cdot}95 \times 10^{-11}$ ($1{\cdot}40 \times 10^{-11}$ to $6{\cdot}19 \times 10^{-11}$)	-4·50 (-9·22 to +0·46)	0·075
Phenytoin	$6{\cdot}01 \times 10^{-1}$ ($1{\cdot}23 \times 10^{-1}$ to $2{\cdot}93 \times 10^{0}$)	$2{\cdot}52 \times 10^{-1}$ ($4{\cdot}73 \times 10^{-2}$ to $1{\cdot}34 \times 10^{0}$)	-9·26 (-15·59 to -2·45)	0·0085
Fosphenytoin	$1{\cdot}51 \times 10^{-9}$ ($2{\cdot}01 \times 10^{-10}$ to $1{\cdot}14 \times 10^{-8}$)	$6{\cdot}02 \times 10^{-10}$ ($9{\cdot}41 \times 10^{-11}$ to $3{\cdot}86 \times 10^{-9}$)	-9·61 (-17·20 to -1·33)	0·024
Trimethadione	$2{\cdot}55 \times 10^{-11}$ ($1{\cdot}27 \times 10^{-11}$ to $5{\cdot}12 \times 10^{-11}$)	$2{\cdot}37 \times 10^{-11}$ ($1{\cdot}22 \times 10^{-11}$ to $4{\cdot}62 \times 10^{-11}$)	-0·81 (-1·09 to -0·53)	<0·0001
Ethosuximide	$6{\cdot}14 \times 10^{-6}$ ($4{\cdot}08 \times 10^{-7}$ to $9{\cdot}25 \times 10^{-5}$)	$6{\cdot}25 \times 10^{-6}$ ($3{\cdot}95 \times 10^{-7}$ to $9{\cdot}90 \times 10^{-5}$)	+0·13 (-10·31 to +11·79)	0·98
Mesuximide	$1{\cdot}32 \times 10^{-10}$ ($3{\cdot}19 \times 10^{-11}$ to $5{\cdot}46 \times 10^{-10}$)	$9{\cdot}18 \times 10^{-11}$ ($2{\cdot}44 \times 10^{-11}$ to $3{\cdot}46 \times 10^{-10}$)	-3·77 (-12·39 to +5·69)	0·42
Clonazepam	$2{\cdot}61 \times 10^{0}$ ($1{\cdot}19 \times 10^{0}$ to $5{\cdot}73 \times 10^{0}$)	$2{\cdot}18 \times 10^{0}$ ($7{\cdot}45 \times 10^{-1}$ to $6{\cdot}36 \times 10^{0}$)	-1·98 (-7·38 to +3·75)	0·49
Carbamazepine	$1{\cdot}01 \times 10^{1}$ ($7{\cdot}91 \times 10^{0}$ to $1{\cdot}30 \times 10^{1}$)	$9{\cdot}85 \times 10^{0}$ ($8{\cdot}00 \times 10^{0}$ to $1{\cdot}21 \times 10^{1}$)	-0·33 (-1·42 to +0·77)	0·55
Oxcarbazepine	$7{\cdot}72 \times 10^{-2}$ ($1{\cdot}07 \times 10^{-2}$ to $5{\cdot}59 \times 10^{-1}$)	$3{\cdot}34 \times 10^{-1}$ ($7{\cdot}07 \times 10^{-2}$ to $1{\cdot}58 \times 10^{0}$)	+17·64 (+2·63 to +34·83)	0·020
Rufinamide	$2{\cdot}88 \times 10^{-8}$ ($2{\cdot}30 \times 10^{-9}$ to $3{\cdot}61 \times 10^{-7}$)	$1{\cdot}25 \times 10^{-6}$ ($8{\cdot}57 \times 10^{-8}$ to $1{\cdot}82 \times 10^{-5}$)	+52·10 (+24·68 to +85·55)	<0·0001
Eslicarbazepine	$1{\cdot}24 \times 10^{-9}$ ($1{\cdot}45 \times 10^{-10}$ to $1{\cdot}07 \times 10^{-8}$)	$1{\cdot}24 \times 10^{-7}$ ($8{\cdot}02 \times 10^{-9}$ to $1{\cdot}91 \times 10^{-6}$)	+66·68 (+31·44 to +111·37)	<0·0001
Valproic acid (valproate)	$1{\cdot}25 \times 10^{1}$ ($9{\cdot}43 \times 10^{0}$ to $1{\cdot}66 \times 10^{1}$)	$1{\cdot}53 \times 10^{1}$ ($1{\cdot}19 \times 10^{1}$ to $1{\cdot}97 \times 10^{1}$)	+2·23 (+1·27 to +3·20)	<0·0001
Valpromide	$1{\cdot}86 \times 10^{-10}$ ($3{\cdot}60 \times 10^{-11}$ to $9{\cdot}65 \times 10^{-10}$)	$9{\cdot}17 \times 10^{-11}$ ($2{\cdot}21 \times 10^{-11}$ to $3{\cdot}80 \times 10^{-10}$)	-7·44 (-14·43 to +0·12)	0·054

Aminobutyric acid	$1{\cdot}36 \times 10^{-9}$ ($1{\cdot}75 \times 10^{-10}$ to $1{\cdot}06 \times 10^{-8}$)	$1{\cdot}48 \times 10^{-9}$ ($1{\cdot}76 \times 10^{-10}$ to $1{\cdot}25 \times 10^{-8}$)	+1·98 (-10·69 to +16·44)	0·77
Vigabatrin	$9{\cdot}30 \times 10^{-5}$ ($7{\cdot}15 \times 10^{-6}$ to $1{\cdot}21 \times 10^{-3}$)	$6{\cdot}99 \times 10^{-4}$ ($6{\cdot}91 \times 10^{-5}$ to $7{\cdot}07 \times 10^{-3}$)	+25·24 (+6·72 to +46·98)	0·0059
Tiagabine	$2{\cdot}17 \times 10^{-8}$ ($1{\cdot}88 \times 10^{-9}$ to $2{\cdot}50 \times 10^{-7}$)	$9{\cdot}90 \times 10^{-10}$ ($1{\cdot}33 \times 10^{-10}$ to $7{\cdot}38 \times 10^{-9}$)	-29·01 (-39·76 to -16·36)	<0·0001
Sultiame	$3{\cdot}14 \times 10^{-9}$ ($3{\cdot}30 \times 10^{-10}$ to $2{\cdot}99 \times 10^{-8}$)	$2{\cdot}28 \times 10^{-8}$ ($1{\cdot}84 \times 10^{-9}$ to $2{\cdot}82 \times 10^{-7}$)	+24·37 (+8·89 to +42·06)	0·0013
Lamotrigine	$2{\cdot}96 \times 10^{0}$ ($1{\cdot}90 \times 10^{0}$ to $4{\cdot}61 \times 10^{0}$)	$5{\cdot}88 \times 10^{0}$ ($4{\cdot}01 \times 10^{0}$ to $8{\cdot}64 \times 10^{0}$)	+7·92 (+6·64 to +9·22)	<0·0001
Felbamate	$4{\cdot}74 \times 10^{-9}$ ($5{\cdot}00 \times 10^{-10}$ to $4{\cdot}50 \times 10^{-8}$)	$4{\cdot}27 \times 10^{-9}$ ($4{\cdot}47 \times 10^{-10}$ to $4{\cdot}08 \times 10^{-8}$)	-1·12 (-13·69 to +13·27)	0·87
Topiramate	$9{\cdot}56 \times 10^{-1}$ ($4{\cdot}44 \times 10^{-1}$ to $2{\cdot}06 \times 10^{0}$)	$1{\cdot}28 \times 10^{0}$ ($5{\cdot}97 \times 10^{-1}$ to $2{\cdot}74 \times 10^{0}$)	+1·18 (-4·86 to +7·60)	0·71
Levetiracetam	$1{\cdot}68 \times 10^{0}$ ($6{\cdot}84 \times 10^{-1}$ to $4{\cdot}15 \times 10^{0}$)	$1{\cdot}10 \times 10^{1}$ ($7{\cdot}84 \times 10^{0}$ to $1{\cdot}53 \times 10^{1}$)	+23·16 (+14·17 to +32·85)	<0·0001
Zonisamide	$9{\cdot}32 \times 10^{-7}$ ($5{\cdot}44 \times 10^{-8}$ to $1{\cdot}60 \times 10^{-5}$)	$3{\cdot}38 \times 10^{-5}$ ($1{\cdot}98 \times 10^{-6}$ to $5{\cdot}78 \times 10^{-4}$)	+49·22 (+22·20 to +82·22)	0·0001
Stiripentol	$2{\cdot}20 \times 10^{-9}$ ($2{\cdot}69 \times 10^{-10}$ to $1{\cdot}80 \times 10^{-8}$)	$1{\cdot}25 \times 10^{-7}$ ($9{\cdot}64 \times 10^{-9}$ to $1{\cdot}61 \times 10^{-6}$)	+56·54 (+25·56 to +95·16)	0·0001
Lacosamide	$3{\cdot}47 \times 10^{-6}$ ($2{\cdot}02 \times 10^{-7}$ to $5{\cdot}95 \times 10^{-5}$)	$5{\cdot}24 \times 10^{-3}$ ($4{\cdot}59 \times 10^{-4}$ to $5{\cdot}98 \times 10^{-2}$)	+124·33 (+69·24 to +197·36)	<0·0001
Retigabine	$1{\cdot}13 \times 10^{-8}$ ($1{\cdot}08 \times 10^{-9}$ to $1{\cdot}19 \times 10^{-7}$)	$2{\cdot}26 \times 10^{-11}$ ($1{\cdot}23 \times 10^{-11}$ to $4{\cdot}15 \times 10^{-11}$)	-51·17 (-61·52 to -38·03)	<0·0001
Perampanel	$5{\cdot}45 \times 10^{-11}$ ($1{\cdot}81 \times 10^{-11}$ to $1{\cdot}64 \times 10^{-10}$)	$1{\cdot}40 \times 10^{-4}$ ($1{\cdot}14 \times 10^{-5}$ to $1{\cdot}71 \times 10^{-3}$)	+409·52 (+270·56 to +600·58)	<0·0001
Brivaracetam	NA	$1{\cdot}08 \times 10^{-5}$ ($6{\cdot}09 \times 10^{-7}$ to $1{\cdot}91 \times 10^{-4}$)	+355·30 (+239·44 to +510·71)	<0·0001
Cannabidiol	NA	$5{\cdot}62 \times 10^{-9}$ ($6{\cdot}21 \times 10^{-10}$ to $5{\cdot}08 \times 10^{-8}$)	+85·15 (+54·72 to +121·56)	<0·0001
Cenobamate	NA	$1{\cdot}45 \times 10^{-10}$ ($3{\cdot}21 \times 10^{-11}$ to $6{\cdot}56 \times 10^{-10}$)	+21·84 (+9·23 to +35·91)	0·0004
Fenfluramine	NA	$4{\cdot}27 \times 10^{-11}$ ($1{\cdot}55 \times 10^{-11}$ to $1{\cdot}17 \times 10^{-10}$)	+8·42 (+0·55 to +16·91)	0·036

[a]Worldwide estimates with 95% CI were calculated by pooling the estimates using meta-analysis (random-effects model).

[b]The multinational trend changes were estimated using linear mixed models, controlling for within-country correlations and assuming the correlations between years were autocorrelated.

[c]Since some DDD/HED are showing very small values, they are presented as three significant figures.

[*]Brivaracetam, Cannabidiol, Cenobamate and Fenfluramine yet to have recorded sales in the database in 2012.

CI = Confidence interval; DDD/HED = Defined daily dose per 100 epilepsy patients per day; NA = Not applicable

Supplementary Table 10. Regional levels, as per WHO classification, of antiseizure medication consumption rate in 2012 and 2022 and average annual percentage change.

Region	DDD/TID in 2012 (95%CI)[a]	DDD/TID in 2022 (95%CI)[a]	Average annual percentage change (%, 95%CI)[b]	P value
**African Region**	50·21 (5·69 to 442·88)	54·92 (10·02 to 301·13)	+1·39 (−0·41 to +3·23)	0·12
**Eastern Mediterranean Region**	14·86 (7·07 to 31·21)	22·86 (11·95 to 43·72)	+4·42 (+0·74 to +8·24)	0·019
**European Region**	76·75 (64·64 to 91·12)	96·80 (83·24 to 112·57)	+2·35 (+1·93 to +2·77)	<0·0001
**Region of the Americas**	35·52 (18·86 to 66·88)	38·56 (20·23 to 73·51)	+0·83 (−1·53 to +3·23)	0·49
**South-East Asia Region**	11·37 (3·04 to 42·50)	17·68 (5·34 to 58·49)	+4·52 (+1·56 to +7·57)	0·0033
**Western Pacific Region**	21·92 (7·56 to 63·55)	30·26 (12·36 to 74·06)	+3·28 (+1·95 to +4·62)	<0·0001

[a]Worldwide and regional estimates with 95% CI were calculated by pooling the estimates using meta-analysis (random-effects model).

[b]The average annual percentage change is calculated using a linear regression model, with log-transformed consumption in DDD/TID as the dependent variable and year as the independent variable. The average annual change was expressed as average annual percentage change, calculated by [exp(the coefficient of the year variable) - 1] x 100%. The multinational and regional trend changes were estimated using linear mixed models, controlling for within-country correlations and assuming the correlations between years were autocorrelated.

[*]Hong Kong, Taiwan and Puerto Rico were excluded in the analysis as they are not included in the WHO region classification
WHO = World Health Organisation; CI = Confidence interval; DDD/TID = Defined daily dose per 10,000 inhabitants per day

Supplementary Figure 1. Multinational antiseizure medications consumption rate from 2012 to 2022.

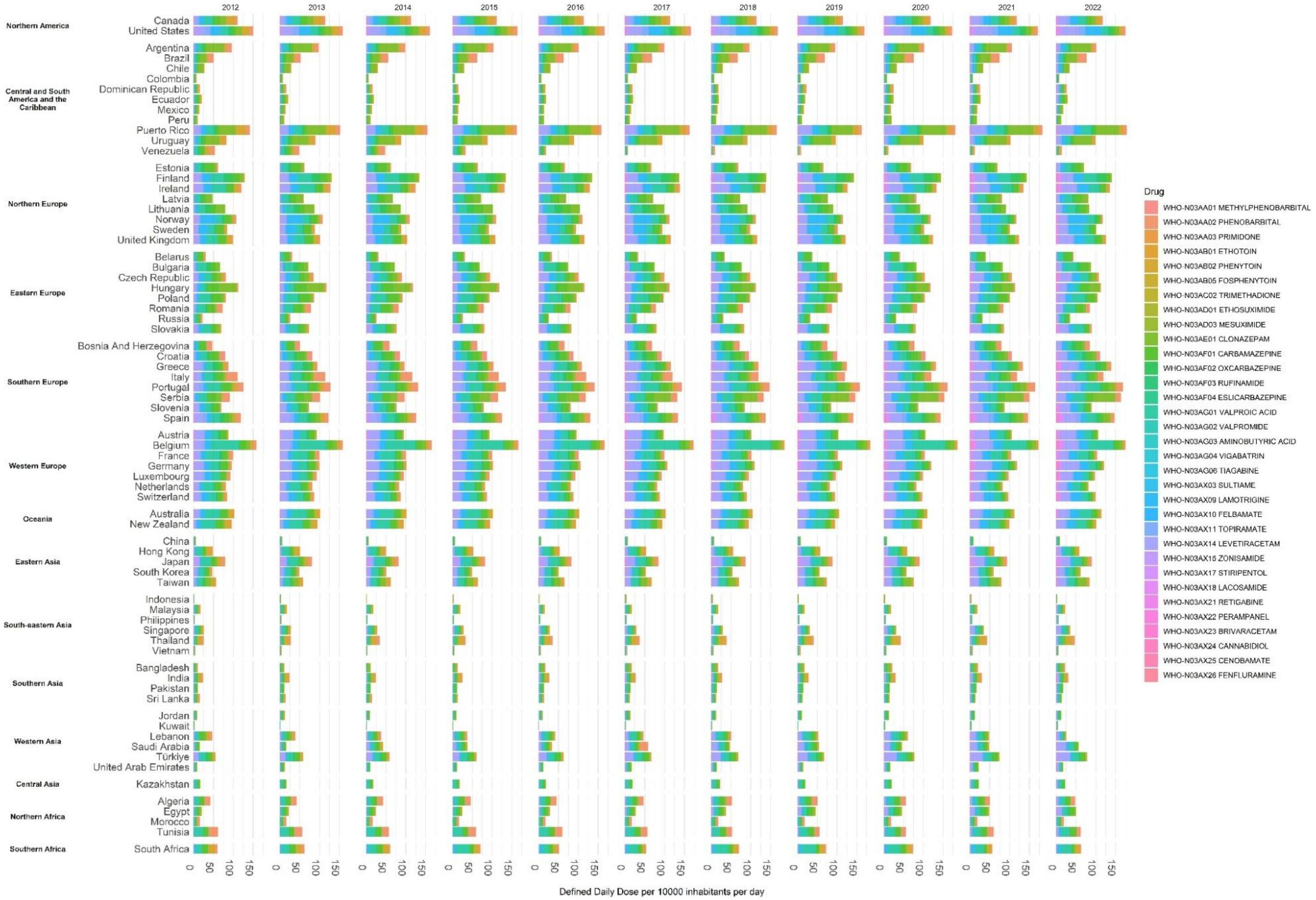

Different colours indicate different antiseizure medications.

Supplementary Figure 2. Top ten multinational antiseizure medication consumption rate from 2012 to 2022.

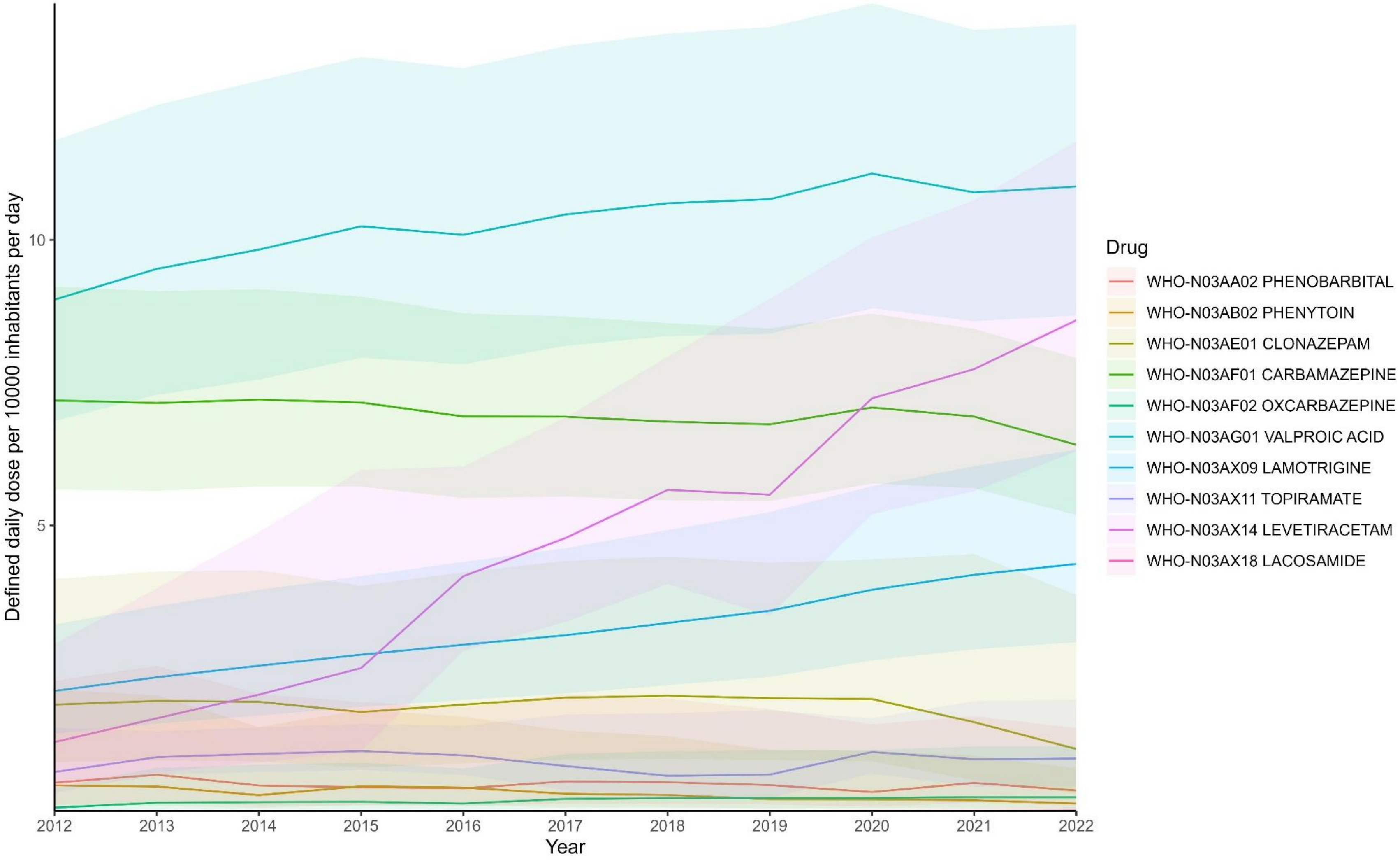

* Please refer to Supplementary table 4 for the multinational consumption rates of top 10 antiseizure medications from 2012 to 2022.

Supplementary Figure 3. Average annual percentage change of valproate consumption rate.

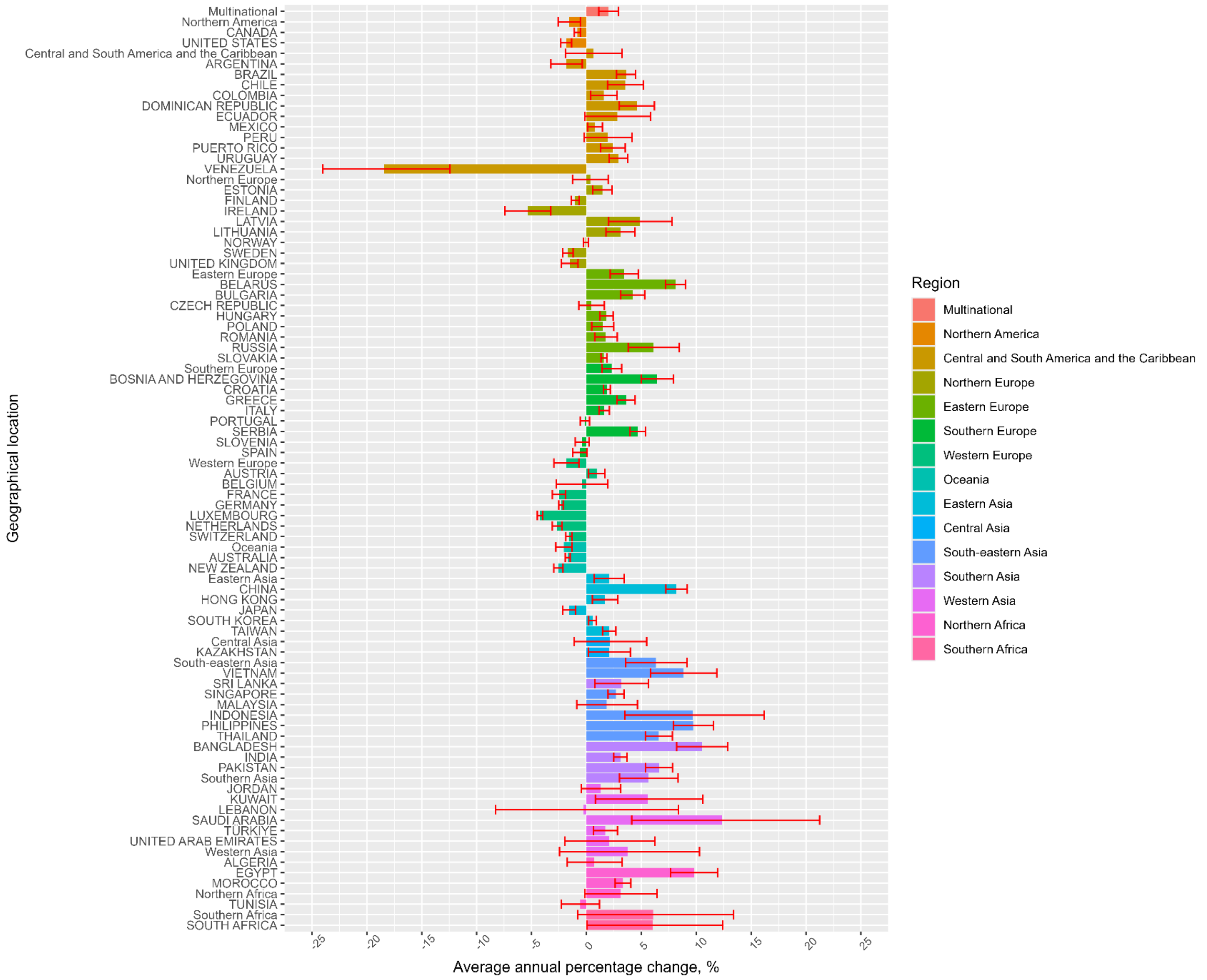

Different colours indicate the geographical regions of the included countries/regions, while the error bars represent the 95% CI for the average annual percentage change between 2012 and 2022.

Supplementary Figure 4. Valproate consumption rate in 2012 and 2022.

2012

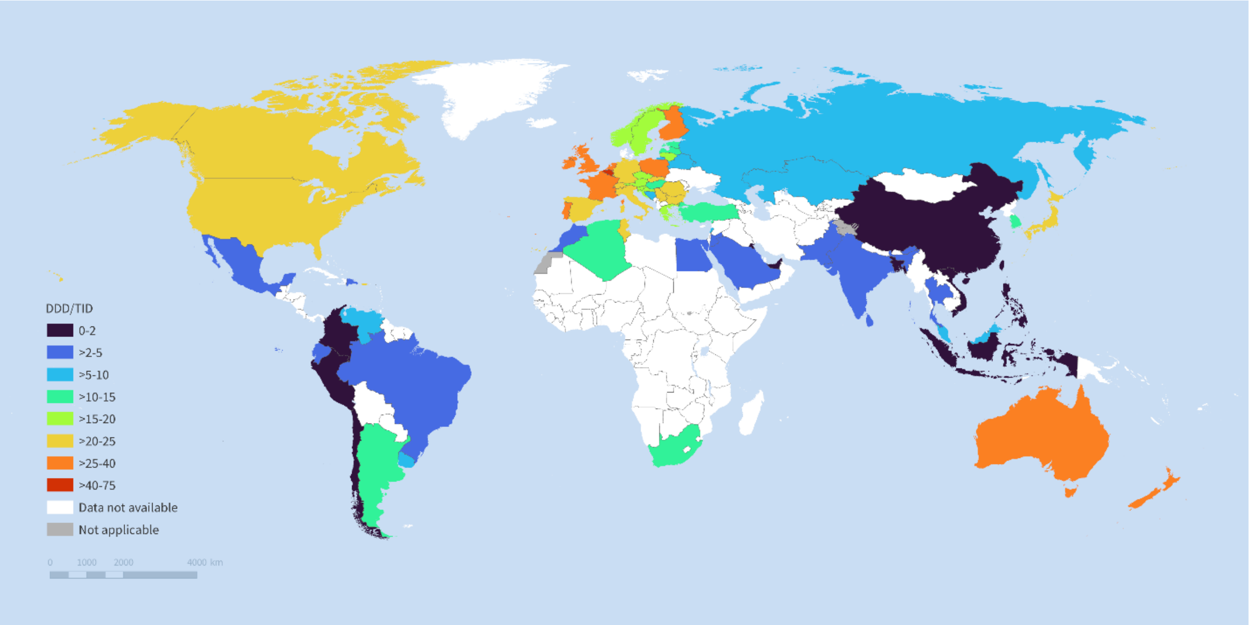

2022

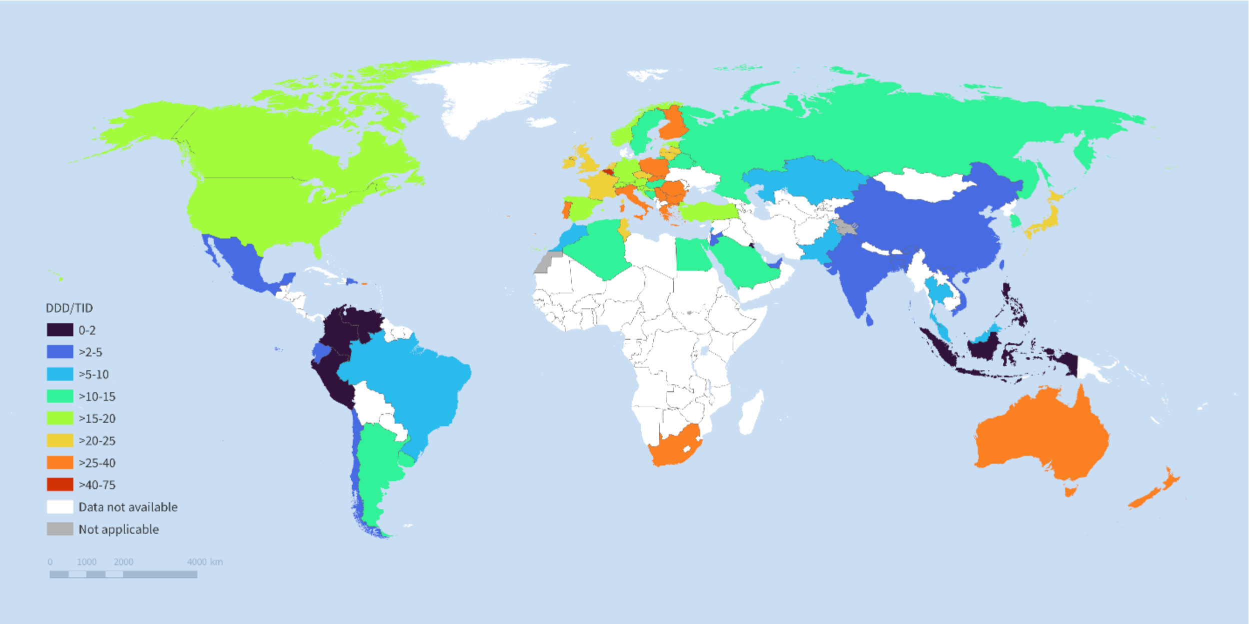

DDD/TID = Defined daily dose per 10,000 inhabitants per day.

* For DDD/TID category thresholds, see the bottom-left legend; both maps use the same breaks and palette. Please refer to Supplementary table 5 for the DDD/TID in each country/region.
